# Pharmacological rescue in patient iPSC and mouse models with a rare DISC1 mutation

**DOI:** 10.1038/s41467-021-21713-3

**Published:** 2021-03-03

**Authors:** Nam-Shik Kim, Zhexing Wen, Jing Liu, Ying Zhou, Ziyuan Guo, Chongchong Xu, Yu-Ting Lin, Ki-Jun Yoon, Junhyun Park, Michelle Cho, Minji Kim, Xinyuan Wang, Huimei Yu, Srilatha Sakamuru, Kimberly M. Christian, Kuei-sen Hsu, Menghang Xia, Weidong Li, Christopher A. Ross, Russell L. Margolis, Xin-Yun Lu, Hongjun Song, Guo-li Ming

**Affiliations:** 1 Department of Neuroscience and Mahoney Institute for Neurosciences, Perelman School for Medicine, University of Pennsylvania, Philadelphia, PA USA; 2 Departments of Psychiatry and Behavioral Sciences, Cell Biology, and Neurology, Emory University School of Medicine, Atlanta, GA USA; 3 Department of Pharmacology, University of Texas Health Science Center at San Antonio, San Antonio, TX USA; 4 Institute for Cell Engineering, Johns Hopkins University School of Medicine, Baltimore, MD USA; 5 Bio-X Institutes, Key Laboratory for the Genetics of Development and Neuropsychiatric Disorders, Shanghai Jiao Tong University, Shanghai, China; 6 Department of Pharmacology, College of Medicine, National Cheng Kung University, Tainan, Taiwan; 7 National Center for Advancing Translational Sciences, National Institutes of Health, 9800 Medical Center Drive, Bethesda, MD USA; 8 Division of Neurobiology, Department of Psychiatry, Johns Hopkins University School of Medicine, Baltimore, MD USA; 9 Department of Neurology, Johns Hopkins University School of Medicine, Baltimore, MD USA; 10 The Solomon H Snyder Department of Neuroscience, Johns Hopkins University School of Medicine, Baltimore, MD USA; 11 Department of Pharmacology, Johns Hopkins University School of Medicine, Baltimore, MD USA; 12 Department of Neuroscience and Regenerative Medicine, Augusta University, Augusta, GA USA; 13 Department of Cell and Developmental Biology, Perelman School for Medicine, University of Pennsylvania, Philadelphia, PA USA; 14 Institute for Regenerative Medicine, University of Pennsylvania, Philadelphia, PA USA; 15 The Epigenetics Institute, Perelman School for Medicine, University of Pennsylvania, Philadelphia, PA USA; 16 Department of Psychiatry, Perelman School for Medicine, University of Pennsylvania, Philadelphia, PA USA; 17 Present Address: Department of Biological Sciences, Korea Advanced Institute of Science and Technology (KAIST), Daejeon, Republic of Korea

**Keywords:** Developmental disorders, Induced pluripotent stem cells

## Abstract

We previously identified a causal link between a rare patient mutation in DISC1 (disrupted-in-schizophrenia 1) and synaptic deficits in cortical neurons differentiated from isogenic patient-derived induced pluripotent stem cells (iPSCs). Here we find that transcripts related to phosphodiesterase 4 (PDE4) signaling are significantly elevated in human cortical neurons differentiated from iPSCs with the DISC1 mutation and that inhibition of PDE4 or activation of the cAMP signaling pathway functionally rescues synaptic deficits. We further generated a knock-in mouse line harboring the same patient mutation in the Disc1 gene. Heterozygous Disc1 mutant mice exhibit elevated levels of PDE4s and synaptic abnormalities in the brain, and social and cognitive behavioral deficits. Pharmacological inhibition of the PDE4 signaling pathway rescues these synaptic, social and cognitive behavioral abnormalities. Our study shows that patient-derived isogenic iPSC and humanized mouse disease models are integral and complementary for translational studies with a better understanding of underlying molecular mechanisms.

## Abstract (web summary)

Previous work has shown in iPSC derived neurons that synaptic impairments are associated with a 4bp DISC1 deletion. Here the authors demonstrate a role for the PDE4 signalling pathway in these synaptic impairments.

## Introduction

Aberrant synaptic development and function have been suggested to be major early pathogenic events for schizophrenia, and significant progress has been made in identifying risk genes for schizophrenia, many of which are associated with synaptic development and function^1,2^. Understanding how various risk genes lead to synaptic deficits and the development of rational therapeutics for mental disorders based on molecular pathology is critically lagging. Human-induced pluripotent stem cells (iPSCs) reprogrammed from patients are promising disease-in-a-dish models to recapitulate disease-relevant cellular and molecular phenotypes for a variety of diseases, including neuropsychiatric disorders^3–7^. Multiple iPSC lines have been derived from idiopathic schizophrenia patients and from mental disorder patients with defined genetic lesions, including a 4-bp deletion in the DISC1 locus, a translocation mutation of DISC1, an exonic deletion of Astrotactin-2 and 15q11.2 microdeletions^8–13^. Human neurons differentiated from these patient-specific iPSC lines were found to exhibit defective synaptic vesicle release, reduced synaptic transmission, and global transcriptomic changes^9,13–15^. In addition to the identification of cellular phenotypes that may be related to causal pathophysiology, iPSC-based models provide opportunities for uncovering disease mechanisms in relevant human cell types, screening for drugs, and developing targeted pharmacological interventions based on mechanistic hypotheses and rational drug design.

While patient iPSC-based models allow for detailed molecular and cellular phenotyping, and the investigation of the underlying mechanisms in a given human cell type, in this system it is not feasible to model behavioral deficits, which are hallmarks of psychiatric disorders. In this study, we started by identifying potential molecular targets in human cortical neurons derived from patient iPSCs carrying a rare DISC1 mutation, followed by testing specific pharmacological treatments to rescue synaptic deficits in these patient-derived neurons. We then generated a humanized mouse model carrying a similar mutation as the patients and showed similar molecular changes and synaptic deficits, as well as deficits in behavioral tests. Finally, we showed that the same pharmacological treatment that was effective in rescuing human neuron synaptic deficits also rescues the synaptic and behavioral deficits in the humanized mouse model.

## Results

### Elevated expression of PDE4 family genes in DISC1 mutant human cortical neurons

**Fig. 1:**
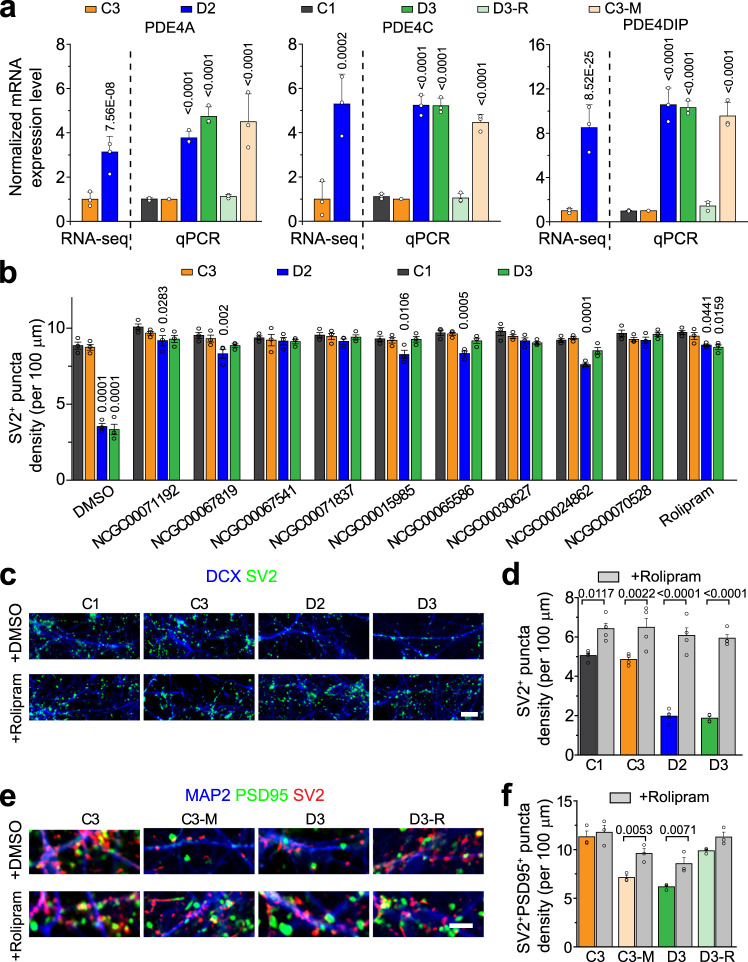
Mechanism-guided pharmacological rescue of synaptic defects of human neurons carrying the DISC1 mutation. **a** Summary of q-PCR validation of RNA-seq results of increased mRNA levels of PDE4A, PDE4C, and PDE4DIP in human forebrain neurons carrying the DISC1 mutation (D2 and D3: neurons derived from patient iPSC lines with DISC1 mutation; C1 and C3: neurons derived from control iPSC lines; C3-M: neurons derived from an isogenic knock-in DISC1 mutant line under the background of the C3 control iPSC line; D3-R: neurons derived from an isogenic DISC1 mutant correction line under the background of the D3 patient iPSC line). Values represent mean ± s.d. (*n* = 3 independent differentiation cultures for qPCR analysis; comparison to C3 using One-way ANOVA with *p* values indicated). **b** Effects on the density of SV2^+^ synaptic puncta in 6-week old forebrain neurons upon treatment with different small molecules. NCGC00071192: 2 µM; NCGC00067819: 2 µM; NCGC00067541: 2 µM; NCGC00071837: 2 µM; NCGC00015985: 10 µM; NCGC00065586: 10 µM; NCGC00030627: 20 µM; NCGC00024862: 20 µM; NCGC00070528: 10 µM; Rolipram: 100 nM. Values represent mean ± s.e.m. (*n* = 3 independent differentiation cultures; comparison to C1 using One-way ANOVA with *p* values indicated). **c**–**d** Representative confocal images of 4-week old neurons from different iPSC lines immunostained with SV2 and DCX upon vehicle (DMSO) or Rolipram (100 nM) treatment. Scale bar, 20 μm (**c**). Also shown is the quantification of SV2^+^ puncta density in neurons derived from different iPSC lines with or without Rolipram treatment (**d**). Values represent mean ± s.e.m. (*n* = 4 independent differentiation cultures; One-way ANOVA with *p* values indicated). **e**–**f** Representative confocal images of 4-week old neurons co-cultured with astrocytes from different iPSC lines immunostained with SV2, PSD95, and MAP2 upon vehicle (DMSO) or Rolipram (100 nM) treatment. Scale bar, 20 μm (**e**). Also shown is the quantification of SV2^+^PSD95^+^ puncta density in neurons derived from different iPSC lines with or without Rolipram treatment (**f**). Values represent mean ± s.e.m. (*n* = 3 independent differentiation cultures; One-way ANOVA with *p* values indicated). Source data are provided as a Source Data file.

Previous RNA-seq analyses of forebrain cortical neurons differentiated from a schizophrenia patient iPSC line with the 4-bp deletion in DISC1 gene (D2 line, Pedigree H; Supplementary Fig. 1a) revealed significant upregulation of multiple transcripts related to the PDE4 family, including PDE4A, 4C, and PDE4DIP (PDE4D interacting protein)^14,16^, when compared to neurons from a family control iPSC line without the DISC1 mutation (C3, Pedigree H) (Fig. 1a). We validated the expression changes with q-PCR and further confirmed with an additional DISC1 mutant line (D3, Pedigree H) from the same family and an additional control line (C1) from outside of the family (Fig. 1a and Supplementary Fig. 1a)^14^. Furthermore, the dysregulated expression pattern of PDE4 family members, including many isoforms of PDE4A, PDE4B, PDE4C, and PDE4D, was observed in the isogenic DISC1 mutation line engineered from the C3 line (C3-M), whereas the isogenic correction line from the D3 mutant line (D3-R) exhibited similar expression levels to control lines^14^ (Fig. 1a and Supplementary Fig. 1b), indicating that the DISC1 mutation is causal for elevated expression of these genes. The protein expression levels of PDE4A, PDE4B, and PDE4C showed corresponding changes to transcripts in neurons from different iPSC lines (Supplementary Fig. 1c–d).

### Rescue of synaptic deficits of DISC1 mutant neurons by PDE4 inhibition

DISC1 has been shown to bind family members of PDE4 phosphodiesterases that negatively regulate cAMP signaling, which in turn plays important regulatory roles in synapse formation and synaptic transmission^17–20^. Similar findings of altered expression of PDE family genes have also been reported in human neurons derived from iPSCs of multiple idiopathic schizophrenia patients^9^ and from patient iPSCs carrying a DISC1 t(1;11) translocation mutation^13^. These results suggest that dysregulation of the PDE pathway might represent a common pathological molecular signature in human neurons derived from patients with schizophrenia and other major mental disorders and could be a potential target for drug development. We evaluated this hypothesis first by phenotypic testing using a small collection of 11 compounds, including 9 active compounds with 7 direct PDE inhibitors and 2 control compounds with similar chemical structures^21^ (Supplementary Table 1). We confirmed the activity and determined the dose responses of these compounds to increase cAMP response element-binding (CREB) protein signaling using two independent reporter assays (Supplementary Fig. 2). Consistent with the previous finding^14^, human forebrain cortical neurons with the DISC1 mutation exhibited reduced SV2^+^ synaptic puncta numbers at 4 weeks after neural differentiation (Fig. 1b, c). Interestingly, all 9 active compounds showed a clear effect in rescuing SV2^+^ synaptic puncta number deficits in neurons differentiated from both D2 and D3 lines (Fig. 1b and Supplementary Fig. 3a). Among the 9 active compounds, NCGC00024862, or Rolipram, is a well-characterized PDE4 inhibitor^22^. We validated the phenotypic results on SV2^+^ synaptic puncta numbers using both patient iPSC lines with Rolipram purchased independently (Fig. 1b–d). Rolipram also rescued the density of SV2/PSD95 paired synaptic puncta of DISC1 mutant neurons using two pairs of isogenic iPSC lines (C3 and C3-M; D3 and D3-R; Fig. 1e, f, and Supplementary Fig. 3c), whereas the two control compounds showed no effect (Supplementary Fig. 3b). Consistent with increased transcript and protein levels of multiple PDE4 family members in mutant neurons, cAMP activity was significantly lower in D3 and C3-M neurons, which can be elevated by the treatment of Rolipram (Supplementary Fig. 3d). We, therefore, focused on Rolipram for further experiments. Rolipram treatment had no effect on the mRNA expression levels of PDE4s (Supplementary Fig. 1b) or cell death under all culture conditions (Supplementary Fig. 3e). Time-course analyses showed that long-term (4 weeks) treatment was needed for a complete rescue of SV2^+^ synaptic puncta numbers (Supplementary Fig. 3f) and the effect was maintained in 6-week old neurons (Supplementary Fig. 3g). Furthermore, treatment with two activators of cAMP-dependent protein kinases, sp-cAMP or forskolin, also showed similar effects in increasing SV2^+^ synaptic puncta numbers in patient neurons (Supplementary Fig. 3h). These results suggest that elevated PDE4 function in DISC1 mutant neurons contributes to aberrant synaptic development.

**Fig. 2:**
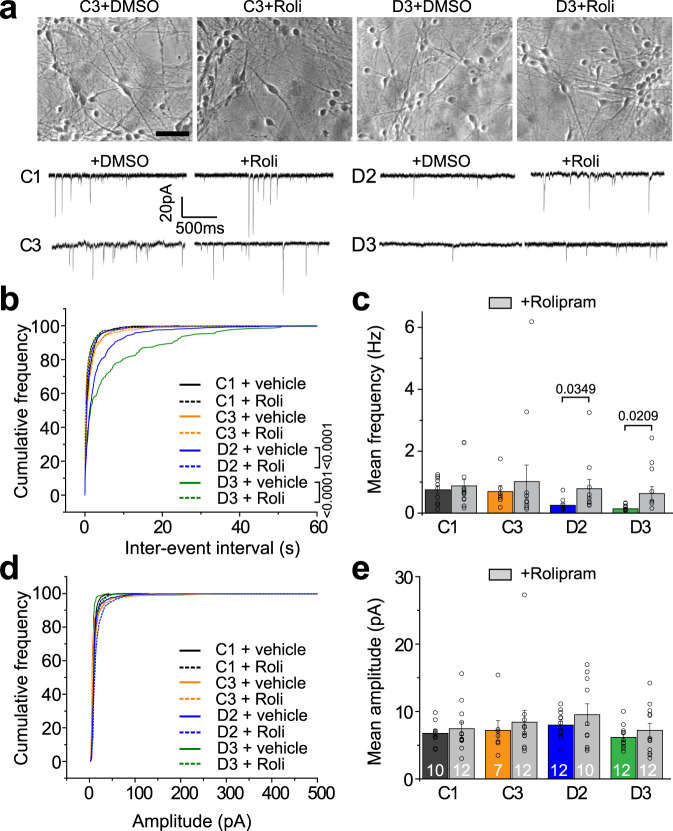
Functional rescue of synaptic defects of DISC1 mutant neurons. **a** Sample images of 6-week-old human neurons differentiated from iPSC lines co-cultured with astrocytes (top) and sample whole-cell voltage-clamp recording traces of excitatory spontaneous synaptic currents (SSCs), with the treatment of vehicle or Rolipram (Roli, 100 nM). Scale bar, 50 μm. The representative images were taken from 3 independent experiments. **b**–**c** Cumulative distribution plots of SSC inter-event intervals (**b**, 4 weeks old neurons) and quantification (**c**, 6 weeks old neurons) showing rescue of reduced SSC frequency of DISC1 mutant neurons after Rolipram treatment. Values in **c** represent mean ± s.e.m. (*n* = 3 independent differentiation cultures; cell number 7 to 12 for each condition; Kolmogorov–Smirnov test for **b**, and One-way ANOVA for **c** with *p* values indicated). **d**–**e** Rolipram treatment has no effect on the amplitude of SSCs. Cumulative distribution plots of SSC amplitude (**d**, 4 weeks old neurons) and quantification (**e**, 6 weeks old neurons). The same neurons as in **b**–**c** were examined. Values in **e** represent mean ± s.e.m. (*n* = 3 independent differentiation cultures; cell number 7 to 12 for each condition). Source data are provided as a Source Data file.

To test whether the rescue of morphological synapses can be translated into functional restoration in DISC1 mutant neurons, we first examined synaptic vesicle release by imaging analysis using Synapto-pHluorin, a genetically encoded optical indicator of synaptic vesicle release and recycling^23^. Compared to control neurons (C3 and D3-R), mutant neurons (D3 and C3-M) exhibited reduced synaptic vesicle release upon depolarizing stimulation with KCl at 4 weeks, which was rescued by Rolipram treatment (Supplementary Fig. 3i). Next, we performed whole-cell recordings of neurons co-cultured on astrocytes at 6 weeks (Fig. 2a). The electrophysiological analysis further confirmed the synaptic transmission deficits in DISC1 mutant neurons, showing reduced frequency, but not amplitude, of miniature excitatory postsynaptic currents (mEPSCs)^14^ (Fig. 2b–e). While Rolipram treatment had a minimal effect on control neurons, the synaptic transmission defects in DISC1 mutant neurons were fully rescued (Fig. 2b–e). Taken together, these results suggest that inhibition of PDE4 activity functionally restores synaptic defects in patient cortical neurons with the DISC1 mutation.

### Increased PDE4 expression and synaptic deficits in the adult brain of humanized Disc1 mutant mice

**Fig. 3:**
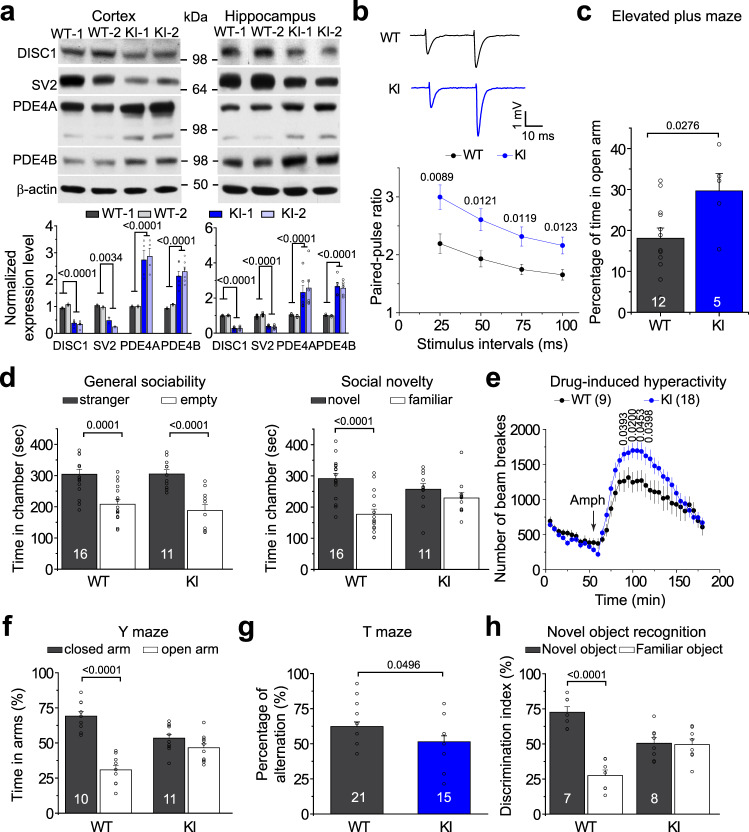
Molecular and behavioral characterization of heterozygous Disc1 KI mice carrying the 4-bp deletion mutation of the Disc1 gene. **a** Protein expression levels in cortical and hippocampal lysates of 4-month-old WT and KI mice. KI refers to heterozygous Disc1 mutant mice. Shown are sample western blot images and quantification of protein levels of DISC1, SV2, PDE4A, and PDE4B. Data were normalized to β-actin for sample loading and then to WT in the same blot for comparison. Values represent mean ± s.e.m. (DISC1: *n* = 6; SV2: *n* = 2; PDE4A: *n* = 4; PDE4B: *n* = 6 independent experiments for cortex; DISC1: *n* = 5; SV2: *n* = 8; PDE4A: *n* = 6; PDE4B: *n* = 5 independent experiments for hippocampus; One-way ANOVA with *p* values indicated). **b** Impaired paired-pulse ratio in Disc1 KI mice. The electrophysiological response of CA1 neurons to paired pulses of stimulation of CA3 neurons from acutely prepared hippocampal slices. The paired pulses were separated by intervals of 25, 50, 75, and 100 ms. Values represent mean ± s.e.m. (*n* = 14 for WT and *n* = 20 for KI; One-way ANOVA with *p* values indicated). **c** Basal anxiety of *Disc1* KI mice in the elevated plus-maze test. The percentage of time spent in the open arm over total time was calculated. Values represent mean ± s.e.m. (*n* = 12 for WT and *n* = 5 for KI, 13-week-old male littermates; One-way ANOVA with *p* values indicated). **d** Three-chamber social preference test showing impaired social novelty recognition of Disc1 KI mice (right), but normal general sociability (left). Time spent in each chamber was calculated. Values represent mean ± s.e.m. (*n* = 16 for WT and *n* = 11 for KI, 16-week-old male littermates; One-way ANOVA with *p* values indicated). **e** Disc1 KI mice showed a higher level of D-amphetamine (Amph)-induced hyperactivity. Locomotor activity was measured 60 min before and 120 min immediately after Amph treatment. A total number of beam breaks was measured. Data are shown in 5 min intervals. Values represent mean ± s.e.m. (*n* = 9 for WT and *n* = 18 for KI, 16-week-old male littermates; One-way ANOVA with *p* values indicated). **f** Impaired spatial working memory of Disc1 KI mice in the Y-maze task. The time spent in each arm during the 5 min test was shown. Values represent mean ± s.e.m. (*n* = 10 for WT and *n* = 11 for KI, 14-week-old male littermates; One-way ANOVA with *p* values indicated). **g** Impaired spontaneous alternation of Disc1 KI mice in the T-maze task. The percentage of alternations (alternated arm entry on two consecutive trials) was scored for 15 trials. Values represent mean ± s.e.m. (*n* = 21 for WT and *n* = 15 for KI, 18-week-old male littermates; One-way ANOVA with *p* values indicated). **h** Decrease of discriminative activity of Disc1 KI mice in the novel object recognition test. KI mice showed no preference for the novel object over the familiar object. Values represent mean ± s.e.m. (*n* = 7 for WT and *n* = 8 for KI, 15-week-old male littermates; One-way ANOVA with *p* values indicated). Source data are provided as a Source Data file.

To complement our in vitro patient iPSC model with an in vivo model for behavioral analyses, we generated a humanized mouse model by knocking in the 4-bp deletion in the Disc1 gene at the same position of exon 12 as identified in mental disorder patients (Supplementary Fig. 4a). The introduced deletion was predicted to generate a truncated form of the DISC1 protein similar to the human mutation (Supplementary Fig. 4b). Surprisingly, the homozygous Disc1 KI mice were embryonic lethal and died before embryonic day 6.5 (Supplementary Fig. 4c). Heterozygous Disc1 KI mice (referred to as KI thereafter) were viable and western blot analysis confirmed the reduced expression level of full-length DISC1 protein in the cortex, hippocampus, and the whole brain, compared to WT littermate controls (Fig. 3a and Supplementary Fig. 4d). Similar reductions in WT DISC1 protein levels have been observed in patient iPSC-derived human neurons with the 4-bp mutation^14^ and with the t(1;11) translocation mutation^13^ of the DISC1 locus. No significant changes in body weight (Supplementary Fig. 4e) or gross brain structure were observed in KI mice (Supplementary Fig. 4f).

We next asked whether the KI mice also show dysregulated expression of PDE4s in the adult brain. Similar to results from the human DISC1 mutant neurons, the adult KI mouse cortex and hippocampus had significantly elevated transcript and protein levels of PDE4s, including both PDE4A and PDE4B (Fig. 3a and Supplementary Fig. 5). Protein levels of SV2, on the other hand, were reduced in both brain regions (Fig. 3a), indicative of potential synaptic deficits. Indeed, cortical neurons cultured from KI mice showed more than a 50% reduction of SV2^+^PSD95^+^ puncta, which can be rescued by Rolipram treatment (Supplementary Fig. 4g). We directly measured synaptic transmission at the well-characterized CA3-CA1 synapses from hippocampal slices acutely prepared from adult animals using paired-pulse facilitation (PPF) protocol, which is commonly used to examine release probability at the presynaptic site^24^. Compared to their WT littermates, the paired-pulse ratio measured in KI mice exhibited a large increase at all stimulus intervals tested, indicating presynaptic deficits (Fig. 3b). Thus, the Disc1 KI mice exhibit changes that are similar to human neurons with the DISC1 mutation at both molecular and electrophysiological levels.

### Behavioral deficits in humanized Disc1 mutant mice

We then asked whether KI mice show any behavioral traits reminiscent of symptomatology associated with schizophrenia and related neuropsychiatric disorders. There were no differences between WT and KI mice in spontaneous locomotor activity in the open field (Supplementary Fig. 6a) and the KI mice did not show depression-related behavior in multiple tests, including two independent sucrose preference tests for anhedonia, the tail suspension test, and forced swim test (Supplementary Fig. 6b–e). The KI mice, however, did show behavioral changes in the elevated plus-maze (EPM) test, which is used as a readout of anxiety-like behavior in mice. KI mice spent more time in the open arms (Fig. 3c), with decreased latency to enter the open arms, a reduced number of protected stretch-attend postures at the closed to open arm threshold, and an increase in unprotected head dips (Supplementary Fig. 6f), indicating a decrease in anxiety-like behaviors in the KI mice.

Deficits in social interaction are closely related to negative symptoms of schizophrenia and are an important early marker of other related mental disorders^25^. To test whether KI mice exhibit altered expression of social behaviors, we performed the three-chamber social interaction test. While no changes in general sociability were observed, KI mice showed clear deficits in social novelty recognition in two independent cohorts of animals (Fig. 3d and Supplementary Fig. 7a). Given that hyperactivity following psychostimulant treatment is a prominent endophenotype in many psychiatric conditions associated with psychosis, we examined the hyperactive response to the psychostimulant D-amphetamine (Amph) in an open field chamber. Acute intraperitoneal (i.p.) injection of Amph induced higher levels of hyperactivity in Disc1 KI mice (Fig. 3e). These data suggest that Disc1 KI mice exhibit social deficits and augmented mesolimbic dopamine function.

Deficits in cognitive function, especially spatial working memory, are considered an endophenotype for schizophrenia^26–28^. We performed two behavioral tests to examine spatial working and short-term memory: a discrete paired-trial variable-delay Y-maze task^29^ and a T-maze spontaneous alternation test. WT mice spent more time in the previously closed arm in the Y-maze test, while KI mice showed no preference between the novel and familiar arms (Fig. 3f), as well as reduced alternations in the T-maze test (Fig. 3g). The novel object recognition test is a non-forced recognition memory test for evaluating memory deficits based on the typical behavior of mice to preferentially explore new objects^30^. In contrast to WT mice spending more time with the novel objects (discrimination index 75%), KI mice showed no preference between the familiar and the novel objects (discrimination index 53%) (Fig. 3h). On the other hand, KI mice did not exhibit behavioral deficits in sensorimotor gating in the pre-pulse inhibition test^31,32^ (Supplementary Fig. 7b) and no differences in fear memory expression between WT and KI were detected in contextual or cued conditioning tests (Supplementary Fig. 7c). Therefore, the KI mice exhibit a set of core behavioral deficits in novelty recognition tests.

### Rescue of synaptic and behavioral deficits of humanized Disc1 mutant mice by PDE4 inhibition

**Fig. 4:**
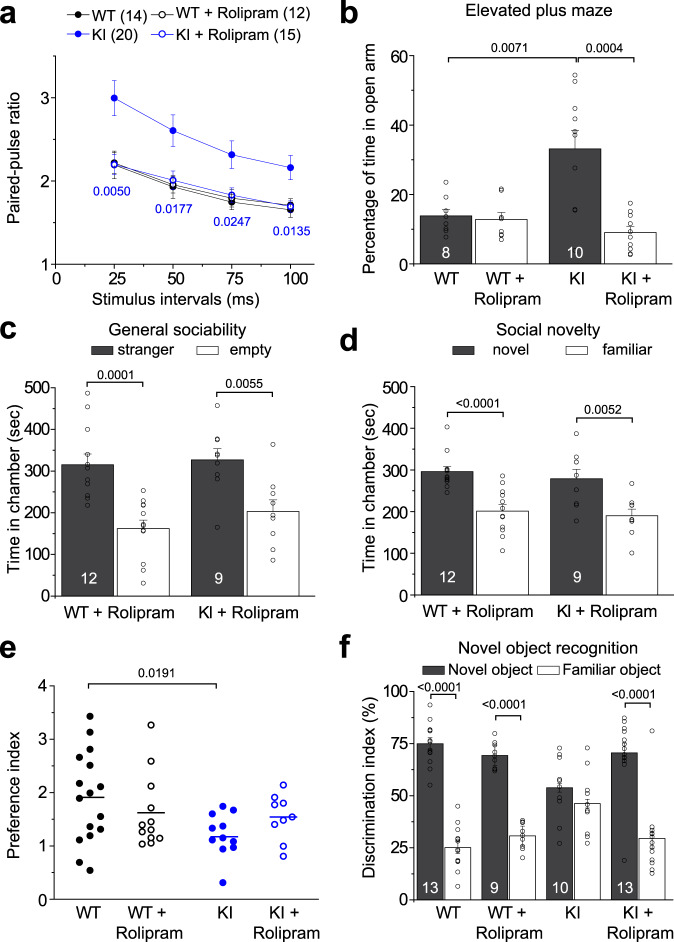
Synaptic and behavioral impairments in Disc1 KI mice rescued by Rolipram treatment. **a** Rolipram restored synaptic transmission deficits in the hippocampus of Disc1 KI mice. Rolipram (0.1 mg/kg) or vehicle was injected 30 min before sacrifice and was present in the bath solution (0.7 μM) throughout the recording. Values represent mean ± s.e.m.; (*n* is indicated in the plot; comparison between KI and KI + Rolipram using One-way ANOVA with p values indicated). The WT and KI without Rolipram treatment from Fig. 3b were replotted here for comparison. **b** Rolipram normalized the anxiety level of Disc1 KI mice in the elevated plus maze test. Values represent mean ± s.e.m. (*n* = 8 for WT and *n* = 10 for KI, 13-week-old male littermates; One-way ANOVA with p values indicated). **c**–**e** Rolipram rescued the social behavioral deficits of Disc1 KI mice in the 3 chambers social interaction test. There was no effect of Rolipram on general sociability (**c**), but Rolipram treatment restored the social novelty recognition of KI mice (**d**). Shown in **e** is a summary of the calculated preference index for novel and familiar mice with or without Rolipram treatment. Values represent mean ± s.e.m. (*n* = 16 for WT, *n* = 13 for WT + Rolipram, = 11 for KI, and *n* = 10 for KI + Rolipram; 16-week-old male littermates; One-way ANOVA with *p* values indicated). **f** Rolipram rescued the cognitive-behavioral deficits of Disc1 KI mice in the novel object recognition test. Values represent mean ± s.e.m. (*n* is indicated in the plot, 15-week-old male littermates; One-way ANOVA with *p* values indicated). Source data are provided as a Source Data file.

Finally, to determine whether altered PDE4 signaling contributes to abnormalities in synaptic transmission and behavioral tests, we first tested basal synaptic transmission by electrophysiological recordings of acute hippocampal slices using the PPF protocol with Rolipram treatment. Rolipram is well tolerated in rodent models and administration of Rolipram has been shown to improve cognitive function in an Alzheimer’s disease mouse model^33,34^. We confirmed the effectiveness of Rolipram in increasing cAMP activity in cultured cortical neurons from both WT and KI mice (Supplementary Fig. 8a), and no effects were observed on the transcriptional levels of PDE4 family members and cell viability (Supplementary Fig. 8b–d). The enhanced paired-pulse ratio observed in KI mice was rescued by Rolipram treatment, while the amplitude of fEPSPs was not affected (Fig. 4a and Supplementary Fig. 8e). We then performed behavioral tests of animals with Rolipram treatment. Rolipram treatment normalized the anxiety level of KI mice in the elevated plus maze test (Fig. 4b). In addition, social novelty and cognitive deficits in KI mice were almost completely rescued by Rolipram treatment (Fig. 4c–f). Together, these results suggest that aberrant PDE4 signaling underlies the synaptic impairments and behavioral deficits in the social and cognitive domains and inhibition of this pathway can reverse those phenotypes.

## Discussion

Although it is controversial whether DISC1 is a general risk gene for schizophrenia, rare DISC1 mutations with high penetrance in specific pedigrees that are linked to schizophrenia and other major mental disorders nevertheless provide an opportunity to understand the molecular and cellular mechanisms underlying disease pathophysiology that may be shared with other genetic risk profiles. Patient-specific iPSC technology provides a unique and robust experimental entry point to harness this opportunity. In this study, using patient iPSC-derived human neurons carrying a rare 4-bp deletion in the DISC1 gene and isogenic neurons as a leading discovery tool, we identified a role of the PDE4-cAMP signaling pathway in synaptic function deficits. Our study provides a direct and mechanistic link between mutant DISC1 and the activity and function of PDE4 at synapses. While previous studies have shown that DISC1 interacts with PDE4^20^, and DISC1 and ATF4 form a transcriptional repressor complex to regulate PDE expression^35^ in heterologous systems, we found elevated expression levels of PDEs in human neurons with the DISC1 mutation and modulation of cAMP signaling does not change their transcriptional levels. We recently showed that DISC1 regulates the binding of ATF4 to its targets and subsequent regulation of downstream gene expression involved in synaptic transmission^36^. DISC1 has also been shown to regulate synaptic function via modulating synaptic vesicle transport^37^ and NMDA receptor dynamics^13^. It would be interesting to investigate in the future whether these phenotypes represent a common feature in patient neurons. Similar molecular changes with transcriptional dysregulation of the PDE4-cAMP signaling pathways have been observed in iPSC-derived neurons from idiopathic schizophrenia patients^9^, from patients with a translocation mutation of DISC1^20^, and in a mouse model with a Disc1 locus impairment^38^. Together, these results suggest that dysregulation of PDE-cAMP signaling might be a potential convergent patho-molecular mechanism underlying schizophrenia and related major mental disorders, and may serve as a target for future drug development. As a proof-of-principle, we tested a small collection of PDE inhibitors, including Rolipram and cAMP pathway activators, and demonstrated their efficacy in reversing the synaptic deficits in human neurons with the DISC1 mutation (Fig. 1). We found that long-term treatment of Rolipram led to a full rescue, but the acute treatment also increased the synaptic density of DISC1 mutant human neurons. The rapid action of Rolipram in modifying synaptic structures is consistent with some previous reports in rodent systems^39,40^.

A previous study has shown that Rolipram treatment rescues the pre-pulse inhibition deficit in the 100P mutant Disc1 mice generated by random mutagenesis that exhibited normal levels of PDE4B^41^. Here we have further established a mouse model carrying the same 4-bp deletion in only one allele of the Disc1 gene, similar to patients with the DISC1 mutation. The KI mice exhibited a spectrum of abnormalities at the molecular level with increased PDE4 signaling, and at the circuitry level with presynaptic transmission deficits, similar to those found in human neurons carrying the DISC1 mutation. At the behavioral level, the KI mice showed limbic dopamine dysregulation, social deficits, and cognitive impairments. Our model shares some similarities, but also shows some differences in behavioral traits compared with previous Disc1 mutant mouse models^41–45^ (Supplementary Table 4). For example, our model did not show elevated movement in the open-field test, increased immobility time in forced swim or tail suspension tests, nor decreased startle responses in pre-pulse inhibition as in other models, but showed deficits in the elevated plus maze, Y and T mazes, and novel subject recognition tests. Together, our mouse model captures a number of features representative of schizophrenia at multiple levels and may be used to better understand disease mechanisms and to develop or validate potential treatments. Indeed, inhibition of PDE4 rescued the synaptic transmission defects and social and cognitive behavioral deficits. Therefore, it is possible to translate treatment efficacy based on in vitro patient iPSC-derived developing human neurons into the rescue of behavioral impairments in adult animals in vivo. It may not be feasible to recapitulate all aspects of a neuropsychiatric disorder using any single model system. Here we demonstrate that complementary model systems of patient-derived isogenic iPSCs and mice with the same disease-relevant genetic lesion may accelerate mechanism-based drug development and cross-species validation of results both in vitro and in vivo.

## Methods

### Human iPSC culture and neural differentiation

All iPSC studies followed institutional IRB protocols approved by Johns Hopkins University School of Medicine and University of Pennsylvania Perlman School of Medicine. Human iPSC lines were previously generated from skin biopsy samples and the isogenic lines were generated by Transcription activator-like effector nucleases (TALEN) as previously described^14^. The pedigree from which these iPSC lines were originally derived has been updated since the previous publication^16^ (Supplementary Fig. 1a). In particular, C3 iPSC lines were derived from subject III9 in the original pedigree, who was initially determined in the 1980s to have a schizotypal personality disorder, but no detail was provided for the rationale for this diagnosis^16^. Re-examination of the subject 20 years later did not detect any evidence of schizotypal personality disorder. Given that personality disorders are stable over time, it was concluded that this subject did not have the schizotypal disorder. All iPSC lines had been fully characterized and were maintained in human iPSC media on mouse embryonic fibroblasts (MEFs). Human iPSC lines were differentiated into forebrain cortical neurons following the previously established protocol^14^. Briefly, iPSC colonies were detached from the feeder layer with 1 mg/ml collagenase and suspended in the embryonic body (EB) medium, consisting of FGF-2-free iPSC medium supplemented with 2 µM Dorsomorphin and 2 µM A-83, on non-treated polystyrene plates for a week to allow the formation of EBs. On day 7, floating EBs were attached to Matrigel-coated 6-well plates in the neural induction medium (NPC medium) containing 2 µM cyclopamine to allow the formation of neural rosettes. On day 22, the rosettes were picked mechanically and transferred to low attachment plates (Corning) to form neurospheres in NPC medium. The neurospheres were then dissociated with Accutase and plated onto matrigel-coated plates/coverslips or a confluent layer of rodent astrocytes as previously described^14^ in neuronal culture medium containing 1 µM cAMP, 200 ng/ml L-Ascorbic Acid, 10 ng/ml BDNF, and 10 ng/ml GDNF.

### Mouse cortical neuron culture

All animal procedures used in this study were performed in compliance with protocols approved by the Institutional Animal Care and Use Committee of Johns Hopkins University, University of Pennsylvania, and University of Texas Health Science Center at San Antonio. Primary cortical neurons were prepared as previously described^46^. Briefly, cortices were dissected from E15.5-17.5 embryos of WT females mated with Disc1 KI male mice, and dissociated neurons were plated on poly-D-lysine-coated coverslips in Neurobasal medium (Gibco) supplemented with 2 mM L-glutamine (Gibco), 100 IU/ml penicillin (Gibco), 100 μg/ml streptomycin (Gibco) and B27 (Gibco). Genotype was determined by PCR with genotyping primers (Supplementary Table 3) from the tail of each embryo. The cell viability assay was performed with the resazurin-based PrestoBlue reagent (ThermoFisher A13261) following the manufacturer’s protocol.

### CRE reporter gene assays

CHO CRE β-lactamase and HEK293 CRE-luciferase cells were plated at 2000 and 2500 cells/well in 5 µl assay medium containing 1% dialyzed FBS in black\clear (β-lactamase cells) and 1% FBS in white\solid (luciferase cells) bottom 1536-well assay plates (Greiner Bio-One North America Inc., Monroe, NC) by a Flying Reagent Dispenser (FRD, Aurora Discovery, San Diego, CA). After culturing overnight, 23 nl of compounds dissolved in DMSO or DMSO alone was added into each well by a Wako Pintool station (Wako, San Diego, CA). The compound treatment was followed by the addition of 1 μl of NKH477 (final concentration, 200 nM for luciferase cells and 10 nM for β-lactamase cells) or media alone in the assay plates by an FRD. For β-lactamase cells, after 3 h incubation at 37 °C, 1 µl per well LiveBLAzer™ FRET B/G CCF4-AM substrate (Life Technologies Corporation, Carlsbad, CA) was added by an FRD and incubated at room temperature for 2 h. Fluorescence intensity at 405 nm excitation and 460 and 530 nm emissions were measured by an Envision plate reader (PerkinElmer, Boston, MA). For luciferase cells, after 4 h incubation at 37 °C, 5 µl per well of ONE-Glo™ reagent (Promega Corporation, Madison, WI) was added and incubated at room temperature for 30 min. Luminescence intensity was measured by a ViewLux plate reader (PerkinElmer).

### qPCR and RNA-seq analyses

Four-week-old human cortical neurons without astrocytes or 14 DIV mouse cortical neurons were used for gene expression analyses. Total RNA was isolated using mirVana kit (Invitrogen) or TRIzol (Invitrogen) according to the manufacturer’s instructions. RNA-seq analysis data are from a previous study^14^. For qPCR, a total of 1 μg RNA was used to synthesize cDNA with the SuperScript® III First-Strand Synthesis System (Invitrogen). Quantitative RT-PCR was then performed using SYBR green (Applied Biosystems) and the QuantStudio™ 6 Real-Time PCR System (Applied Biosystems) or the StepOnePlus™ Real-Time PCR System (Applied Biosystems). Expression levels for all genes were normalized to the housekeeping gene GAPDH and expressed relative to the relevant control samples. Primers used for this study are summarized in Supplementary Table 3.

### Immunocytochemistry and immunohistochemistry

Four-week-old or six-week-old human neurons or 14 days in vitro (DIV) mouse cortical neurons were fixed with 4% paraformaldehyde (Sigma) for 15 min at room temperature. Samples were permeabilized and blocked with 0.25% Triton X-100 (Sigma) and 10% donkey serum in PBS for 20 min as previously described^14^. Samples were then incubated with primary antibodies at 4 °C overnight, followed by incubation with secondary antibodies for 1 h at room temperature. Images were taken by a Nikon Eclipse Ti-E or Zeiss LSM 800 Airyscan confocal microscope. For human neurons, images were acquired from three or four independent cultures with identical settings for parallel cultures. In each culture, 25 fields (600 × 600 µm) of images were taken using a ×20 objective, and 25–30 neurons from each field were analyzed with ImageJ (NIH). For mouse neurons, 60 µm × 60 µm size of images were acquired with a ×63 objective and identical settings for parallel cultures. For analysis of synaptic bouton density, human neurons were cultured on a confluent layer of rodent astrocytes at a density of 1.25 × 105 cells/well in 24-well plates as previously described^14^. Mouse cortical neurons were plated at a density of 2.5 × 10^5^ cells/well of 24 well plate and cultured for 6 days in vitro. Then neuronal cultures were treated with Rolipram (100 nM) for 8 days (total 14 days culture in vitro) and fixed with 4% PFA after washing with PBS. Numbers of SV2/PSD95 pairs were manually counted. Synaptic density was determined by D (D = total SV2^+^ puncta or SV2/PSD95 pair per 100 µm (human) or per 20 µm (mouse) total DCX^+^ dendritic length). For quantification, 24 images (WT DMSO), 26 images (WT Rolipram), 48 images (KI DMSO), and 39 images (KI Rolipram) from 3 independent cultures of mouse primary cortical neurons were used. In a subset of analyses, we also quantified the density of SV2/PSD95 pair per 10^4^ μm^2^ MAP2^+^ dendritic area for human neurons (Supplementary Fig. 3c). Detailed information about primary antibodies is summarized in Supplementary Table 2.

### cAMP activity measurements

the cAMP activity was measured with a competition enzyme-linked immunoassay kit according to the manufacturer’s protocol (Cell Signaling #4339). In this assay, HRP-conjugated cAMP competes with cAMP from neuronal lysates for binding to a cAMP-specific antibody. For mouse cortical neurons, 2.5 × 10^5^ primary cortical neurons were plated on each well of 24 well plates and cultured for 14 days in vitro. For human iPSC-derived forebrain neurons, 1.25 × 105 of human neurons were plated on each well of 24 well plates and cultured for 4 weeks in vitro. Then neuronal cultures were treated with Rolipram (100 nM) for 15 min and lysed with Cell Lysis Buffer (provided by kit) after washing with PBS. Purified cAMP provided by the kit (Cell Signaling #4339) was diluted and used for setting the standard curve.

### Synapto-pHluorin imaging of human forebrain neurons

pLenti-Synapsin-pHoenix-WPRE was a gift from Christian Rosenmund (Addgene plasmid # 70111; http://n2t.net/addgene:70111; RRID:Addgene_70111). High-titer lentivirus was produced from HEK293 cells and used to infect 3-week-old human iPSC-derived forebrain neurons cultured on glass coverslips in 24-well plates. One week later, neurons were used for time-lapse live imaging for monitoring synaptic vesicle release. Briefly, human iPSC-derived neurons were first transferred to image buffer (in mM), consisting of 170 NaCl, 3.5 KCl, 0.4 KH2PO_4_, 5 NaHCO_3_, 1.2 Na_2_SO_4_, 1.2 MgCl_2_, 1.3 CaCl_2_, 5 glucose, 20 N-tris(hydroxymethyl)-methyl-2-aminoethane-sulfonic acid, pH 7.4, ∼360 mOsmol. Live imaging was then performed on a Nikon Eclipse Ti-E imaging system with a 20× NA 0.75 Plan Apochromat Lambda objective. Excitation (470/40 nm) and emission (525/50 nm) filters for EGFP were used. Neurons were perfused with imaging buffer for 30 s as the baseline, followed by stimulation with 60 mM KCl for 2 min. Images from three independent cultures were acquired every 3 s with identical settings for all conditions through a Nikon DS-Qi2 sCMOS Camera with the use of Nikon NIS-Elements Software. In each culture, 10 images (600 × 600 µm) were taken. Background-subtracted images were then analyzed by creating a region of interest (Diameter: 10 pixels) that circumscribed each pHluorin^+^ synaptic vesicle and analyzed using NIH ImageJ software with the Time Series Analyzer plugin. The normalized peak of pHluorin signal was determined by ∆F_peak_ (∆F_peak_ = [(F_peak _− B_peak_) − (F_baseline _− B_baseline_)]/(F_baseline _− B_baseline_)).

### Electrophysiological analyses

Whole-cell recordings of human neurons were performed using a Multiclamp 700A patch-clamp amplifier (Molecular Devices, Palo Alto, CA) as previously described^14^. Data were acquired using pClamp 9 software (Molecular Devices, Palo Alto, CA), sampled at 10 kHz and filtered at 1 kHz. Spontaneous synaptic events were analyzed using MiniAnalysis software (Synaptosoft, Decator, GA). All experiments were conducted at room temperature.

PPF recordings of mouse hippocampal slices were performed on acutely prepared brain slices. Briefly, 400 μm thick slices were cut using a vibratome (Leica VT1200S) and transferred to a chamber containing the external solution (in mM: 125.0 NaCl, 2.5 KCl, 1.3 KH_2_PO_4_, 25.0 NaHCO_3_, 2 CaCl_2_, 1.3 MgCl_2_, 1.3 sodium ascorbate, 0.6 sodium pyruvate, 10 dextrose, pH 7.4, 320 mOsm), bubbled with 95% O_2_/5% CO_2_. Electrophysiological recordings were obtained at 32 °C–34 °C. To record PPF, a glass pipette filled with 1 M NaCl was placed in the CA1 stratum radiatum, and a bipolar stimulating electrode was placed along the Schaffer collateral fibers. The stimulation strength was set to elicit responses with an amplitude that was 30-40% of the maximum. Paired pulses were separated by intervals of 25, 50, 75, and 100 ms at baseline stimulation intensity. The PPF was determined by measuring the percentage increase in the peak amplitude of the second response, taking the first response as 100%. For drug administration, the mice were injected with a single dose of Rolipram (0.1 mg/kg body weight) 30 min before slice preparation^34^. Rolipram (0.7 μM) was also present in the bath solution throughout the entire experiment. Data were collected using an Axon 200B amplifier and acquired via a Digidata 1322 A (Axon Instruments) at 10 kHz, and analyzed using pClamp software.

### Generation of a knock-in mouse line with a 4-bp deletion in the Disc1 locus

The targeting vector was designed to replace exon 12 of the Disc1 gene with a 4-bp deleted exon 12 and both a positive selection marker (PGK promoter-driven neomycin-resistant gene) and a negative selection marker (PGK promoter-driven thymidine kinase gene) were appended to the construct to select against non-homologous recombination (Supplementary Fig. 4a). To generate 4-bp deleted exon 12, 687-bp including exon 12 was synthesized in vitro, and 5’ and 3’—flanking arms were amplified by PCR from the C57BL/6 BAC clone. The targeting vector was linearized and electroporated into C57BL/6 mouse embryonic stem cells (Gene Targeting and Transgenic Mouse Models Core in the University of Cincinnati), and homologous recombination was confirmed by PCR screening using a designed primer set. Targeted clones were injected into C57BL/6 albino (C57BL/6J-Tyr^c-2J^) blastocysts, which were subsequently transferred into pseudopregnant foster mothers. Confirmation of germ-line transmission of the 4-bp deletion allele was performed by PCR genotyping with ear DNA of black pups from a chimera and C57BL/6 albino breeding pair. To avoid potential alteration of gene expression or splicing caused by the existence of the PGK-Neo cassette, the cassette was deleted by crossing with the CMV-Cre mouse (The Jackson Laboratory, Bar Harbor, ME, USA). The sequences of genotyping primers are in Supplementary Table 3. This mouse line will be available from The Jackson Laboratory (Stock No. 036106).

### Western blot analysis

Hippocampal, cortical, or whole-brain tissues were quickly dissected out from 4-month-old mice and homogenized in RIPA buffer (50 mM Tris pH 7.5, 120 mM NaCl, 1% Triton X-100, 0.5% Sodium Deoxycholate, 0.1% SDS, 5 mM EDTA, 10 mM NaF supplemented with protease inhibitor (Invitrogen)). 4-week-old human iPSC-derived forebrain neurons were harvested for western blot analyses. Cells were lysed in RIPA buffer (150 mM NaCl, 1% Triton X-100, 0.5% sodium deoxycholate, 0.1% SDS; 50 mM Tris, pH 8.0) containing Complete Protease Inhibitor Cocktail (Roche). Samples were left on ice for 30 min and sonicated briefly. The insoluble fraction was removed by centrifugation at 18,000 × *g* for 15 min at 4 °C. Protein concentration was determined by BCA protein assay kit (Bio-Rad). 2× SDS sample buffer (Bio-Rad) containing 5% β-mercaptoethanol (Sigma) was added to equal amounts of protein. Proteins were then separated by 4-15% SDS PAGE (Bio-Rad) and transferred to PVDF (0.2 μm) or nitrocellulose membrane (0.45 μm). 5% dried milk in TBST (Tris-buffered saline with 0.1% Tween 20) was incubated for blocking, and membranes were applied with specific antibodies as listed in Supplementary Table 2. After washing with TBST and incubation with horseradish peroxidase-conjugated anti-rabbit or anti-mouse IgG (Santa Cruz Biotechnology), the antigen-antibody was detected by chemiluminescence (ECL; Pierce), and X-ray film (GE Healthcare). The full-length gel images are included in the source data.

### Behavioral analysis

The animals were kept at an ambient temperature of 21 °C and humidity of 40–60% under a 12-h light/dark cycle with a standard chow diet. Behavioral tests were performed with age-matched littermates of 10-week-old to 24-week-old animals of two independent cohorts at two institutions: Johns Hopkins University (JHU) in Dr. Guo-li Ming’s Lab and University of Texas Health Science Center San Antonio (UTSA) in Dr. Xin-yun Lu’s lab. All the behavioral tests were performed with male mice.

For behavior tests with drug treatment, Rolipram (0.1 mg/kg body weight) or PBS was injected intraperitoneally (i.p.) 30 min before the test. All behavior tests were completed within 1 h after Rolipram treatment.

#### Open field test for locomotor activity

(UTSA) Mice were placed in Superflec Fusion open field cages (40 × 40 × 40 cm, Omnitech Electronics Inc, Columbus, OH) and allowed to freely explore for 120 min. The movements of mice, including distance traveled and vertical activity (rearing), were monitored by infrared photosensors equipped on the cage and the total distance traveled was analyzed through Fusion software (OmniTech Electronics, Columbus, OH, USA).

#### Elevated plus-maze test

JHU—Mouse behavior on the elevated plus-maze was recorded by a CCD camera positioned directly above the elevated plus-maze for 5 min. The time spent on the open and closed arms and the number of entries made into each arm were scored. An entry was defined as all four paws being positioned within one arm. The degree of anxiety was assessed by the percentage of time spent in an open arm (time spent in the open arms/total time spent in all arms).

(UTSA) The plus maze consisted of four arms (30-cm long and 5-cm wide) arranged in the shape of a “plus” sign and elevated to a height of 70 cm from the floor. Two arms had no side or end walls (open arms), and the other two arms had side walls and end walls (12-cm high) but were open on top (closed arms). The arms intersected at a central platform (5 × 5 cm) that allowed access to all of the arms. Mice were placed in the central square facing the corner between a closed arm and an open arm and allowed to explore the elevated plus-maze for 5 min. Mouse behaviors on the elevated plus-maze were recorded by a CCD camera positioned directly above the maze. The time spent on the open and closed arms and the numbers of entries made into each arm were scored. Entry was defined as all four paws entering one arm. Anxiety-like behavior was assessed by calculating the percentage of open arm entries (entries into the open arms/total entries into all arms) and percentage of open arm time (time spent in the open arms/total time spent in all arms).

#### Forced swim test

JHU—The experiment was performed in a glass cylinder (20 cm high × 13.5 cm diameter) filled with water (23 ± 2 °C) to a 12 cm depth. Mice were allowed to swim for 6 min, and total time spent immobile, defined as the absence of struggling and floating motionless in the water, and the latency to the first immobile episode was counted during the last 5 min of the test.

#### Tail suspension test

(UTSA) The tail suspension apparatus consisted of a wooden box (30 × 30 × 30 cm) with an open front. A horizontal bar was placed 1.0 cm from the top with an attached vertical bar hanging down from the center. Mice were individually suspended to the vertical bar with the adhesive tape fixed 2.0 cm from the tip of the tail. A charge-coupled device camera was positioned in front of the tail suspension test box and behavior was recorded for 6 min. The apparatus was cleaned with 20% ethanol between trials. Immobility was defined as the absence of any movements except those caused by respiration.

#### Sucrose preference tests

(UTSA) Operant chamber test: The sucrose preference test was performed in operant chambers (Med Associates) equipped with computerized lickometers used to count every tongue contact made (‘licks’) with either 1% sucrose solution or plain water. Mice were habituated to the operant chamber and water-deprived overnight before testing. Mice were offered a choice between sucrose solution and water and the lickometer recorded the number of licks to each sipper tube. The preference was calculated as the number of licks to the saccharin sipper tube divided by the total number of licks to all the sipper tubes times 100.

(UTSA) Two bottle choice test: Mice were habituated to drink from two bottles of water before testing. To measure the preference for sucrose solution, the animals were singly housed and tested with a free choice of one bottle of water and one bottle of 1% sucrose for 4 days. Water and sucrose intake was measured daily and the position of the two bottles was switched every day. Sucrose preference for 1% sucrose solution was expressed as a percentage of the volume of sucrose solution intake relative to the volume of total fluid intake.

#### Sociability and social novelty tests

(UTSA) Sociability and social novelty tests were performed in a standard mouse cage separated into three chambers by partitions. Each chamber measures 20 cm (length) × 40 cm (width) × 22 cm (height). Each side chamber contained a cylindrical wire cage to enclose a live mouse. Before the test, the test mouse was placed in the center of the empty three-chamber box for habituation. The mouse was allowed to freely explore each chamber for 15 min. In session 1, an age-matched and sex-matched C57BL/6 mouse (S1) that had never been exposed to the test mouse was placed in one of the two-wire cages. The wire cage on the other side remained empty. In session 2, a second age-matched and sex-matched C57BL/6 stranger mouse (S2, novel) that had never been exposed to the test mouse was placed in the wire cage that had been empty in the previous session. The test mouse was placed in the center and allowed to freely explore all chambers for 10 min for each session, able to choose between the familiar or the stranger mouse. The movement of the mouse was recorded by a video camera controlled by AnyMaze software (San Diego Instruments Inc., San Diego, CA, USA) and the time spent in each chamber was calculated.

JHU—The same behavior test was performed, but the interaction time with a familiar or stranger mouse was calculated.

#### Drug-induced hyperactivity test

JHU—For measurement of D-amphetamine-induced hyperactivity, saline was intraperitoneally (i.p.) administered 30 min after habituation to the test cage as a control for the effect of the injection, D-amphetamine (0.3 mg/kg, Dainippon Sumitomo Pharma Co. Ltd., Osaka, Japan) was then i.p. administered 30 min after saline injection. Locomotor activity was measured for 120 min.

#### Acoustic startle response and prepulse inhibition tests

JHU—Experiments were performed using a startle chamber (San Diego Instruments Inc., San Diego, CA, USA). Presentations of the acoustic stimuli were controlled by the SR-LAB software and interface system, which also rectified, digitized and recorded responses from the accelerometer. To measure the acoustic startle response, each mouse was kept in the chamber on a movement-sensitive platform for 5 min with 70 dB background white noise for acclimation. Then 10 startle stimuli (40 ms white noise, 120 dB) were delivered every 20 s.

During each PPI session, a mouse was exposed to the following types of trials, a pulse-alone trial (a 120-dB, 100-ms), a null trial (no-stimulus trial), and five pre-pulse trials (74 dB, 78 dB, 82 dB, 86 dB, and 90 dB). Each session consisted of six presentations of each type of trial presented in a pseudorandom order. PPI was assessed as the percentage scores of PPI (%PPI): 100 (mean startle amplitude on pulse-alone trials + mean startle amplitude on prepulse – pulse trials/mean startle amplitude on pulse-alone trials) for each animal separately.

#### Y-maze

JHU—Mice were placed into one of the arms of the maze (start arm) and allowed to explore the maze with one of the arms closed for 15 min (training trial). After a 1-h intertrial interval, mice were returned to the Y maze and placed in the start arm. Then, the mice were allowed to explore freely all three arms of the maze for 5 min (test trial). The time spent in each arm was automatically calculated by AnyMaze software (San Diego Instruments Inc., San Diego, CA, USA).

#### T-Maze spontaneous alternation

(UTSA) The T-maze consisted of a start arm (35 × 10 cm) and two identical goal arms (35 × 10 × 16 cm) arranged in the form of a T. At the beginning of the start arm, a removable wooden door separated a 10-cm-long start box from the rest of the arm. The test session consists of one forced trial and 14 subsequent free-choice trials. In the first trial, the mouse was placed in the start box and after 5 s of confinement, the door was lifted and the mouse allowed to explore the start arm and one of the goal arms. At this point, entry to the other goal arm was blocked. During 14 ‘free-choice’ trials, the mouse was able to choose freely between the left and right goal arms. After the door of the start arm was opened, the mouse was free to choose between both goal arms (all doors open). As soon as the mouse entered one goal arm (including all four feet and the tail tip), the other goal arm was closed. The mouse was returned to the closed start box, and the next free-choice trial was started after a 5-s confinement. The T-maze was thoroughly cleaned with a 20% ethanol solution before each new mouse started its session. The consecutive choices made by the mice were recorded. The overall alternation rate was calculated as a percentage (100 × number of alternations/total number of choices).

#### Novel object recognition test

JHU—Mice were habituated to an open box (30 × 30 × 35 high cm) for two days. During the training session on the third day, two novel objects were placed in the open box, and the animals were allowed to explore for 10 min. In the retention session, the animals were placed into the same box with one of the familiar objects and one novel object. The mice were then allowed to explore freely for 5 min. The preference index during the retention session and the ratio of the amount of time spent exploring the novel object over the total time spent exploring both objects were measured.

#### Fear conditioning tests

JHU—The mice were placed in the test chamber and allowed to freely explore during a 120 s habituation period.

Tone dependent conditioning test: A tone (an auditory cue) was then presented at 2k Hz for 30 s. A foot shock (0.75 mA) was administered during the last 2 s of the tone followed by a 60 s interval period and then two more pairings of the tone and shock. An hour later, the freezing response for the tone was measured for 300 s in a different chamber with distinct size, floor texture, and the odor was analyzed.

Contextual conditioning tests were performed 24 hrs later with the same cohort. The mice were placed in the training chamber without tone cues. The freezing response to the context was analyzed.

### Reporting summary

Further information on research design is available in the Nature Research Reporting Summary linked to this article.

## Supplementary information

Supplementary Information

Reporting Summary

## Data Availability

The authors declare that all data supporting the findings of this study are available within the paper and its supplementary information files. Source data are provided with this paper.

## Source data

Source Data
