# Author Correction: Pharmacological rescue in patient iPSC and mouse models with a rare DISC1 mutation

**DOI:** 10.1038/s41467-021-23263-0

**Published:** 2021-04-30

**Authors:** Nam-Shik Kim, Zhexing Wen, Jing Liu, Ying Zhou, Ziyuan Guo, Chongchong Xu, Yu-Ting Lin, Ki-Jun Yoon, Junhyun Park, Michelle Cho, Minji Kim, Xinyuan Wang, Huimei Yu, Srilatha Sakamuru, Kimberly M. Christian, Kuei-sen Hsu, Menghang Xia, Weidong Li, Christopher A. Ross, Russell L. Margolis, Xin-Yun Lu, Hongjun Song, Guo-li Ming

**Affiliations:** 1 Department of Neuroscience and Mahoney Institute for Neurosciences, Perelman School for Medicine, University of Pennsylvania, Philadelphia, PA USA; 2 Departments of Psychiatry and Behavioral Sciences, Cell Biology, and Neurology, Emory University School of Medicine, Atlanta, GA USA; 3 Department of Pharmacology, University of Texas Health Science Center at San Antonio, San Antonio, TX USA; 4 Institute for Cell Engineering, Johns Hopkins University School of Medicine, Baltimore, MD USA; 5 Bio-X Institutes, Key Laboratory for the Genetics of Development and Neuropsychiatric Disorders, Shanghai Jiao Tong University, Shanghai, China; 6 Department of Pharmacology, College of Medicine, National Cheng Kung University, Tainan, Taiwan; 7 National Center for Advancing Translational Sciences, National Institutes of Health, 9800 Medical Center Drive, Bethesda, MD USA; 8 Division of Neurobiology, Department of Psychiatry, Johns Hopkins University School of Medicine, Baltimore, MD USA; 9 Department of Neurology, Johns Hopkins University School of Medicine, Baltimore, MD USA; 10 The Solomon H Snyder Department of Neuroscience, Johns Hopkins University School of Medicine, Baltimore, MD USA; 11 Department of Pharmacology, Johns Hopkins University School of Medicine, Baltimore, MD USA; 12 Department of Neuroscience and Regenerative Medicine, Augusta University, Augusta, GA USA; 13 Department of Cell and Developmental Biology, Perelman School for Medicine, University of Pennsylvania, Philadelphia, PA USA; 14 Institute for Regenerative Medicine, University of Pennsylvania, Philadelphia, PA USA; 15 The Epigenetics Institute, Perelman School for Medicine, University of Pennsylvania, Philadelphia, PA USA; 16 Department of Psychiatry, Perelman School for Medicine, University of Pennsylvania, Philadelphia, PA USA; 17 Present Address: Department of Biological Sciences, Korea Advanced Institute of Science and Technology (KAIST), Daejeon, Republic of Korea

**Keywords:** Developmental disorders, Induced pluripotent stem cells

Correction to: *Nature Communications* 10.1038/s41467-021-21713-3, published online 3 March 2021.

The original version of this Article contained an error in the spelling of the author Srilatha Sakamuru, which was incorrectly given as Srilatha Salamuru. This has now been corrected in both the PDF and HTML versions of the Article.

