# Supplementary material for: Pharmacological rescue in patient iPSC and mouse models with a rare DISC1 mutation
Source: Nat Commun. 2021 Mar 3;12:1398. doi: 10.1038/s41467-021-21713-3 (PMC7930023; doi:10.1038/s41467-021-21713-3)
Supplement: Supplementary file 1 — Supplementary Information [file 41467_2021_21713_MOESM1_ESM.pdf]

Supplementary Information

Pharmacological rescue in patient iPSC and mouse models with a rare DISC1 mutation

Nam-Shik Kim, Zhexing Wen, Jing Liu, Ying Zhou, Ziyuan Guo, Chongchong Xu, Yu-Ting Lin, Ki-Jun Yoon, Junhyun Park, Michelle Cho, Minji Kim, Xinyuan Wang, Huimei Yu, Srilatha Sakamuru, Kimberly M. Christian, Kuei-sen Hsu, Menghang Xia, Weidong Li, Christopher A. Ross, Russell L. Margolis, Xin-Yun Lu, Hongjun Song, and Guo-li Ming

The supplementary information contains:
Supplementary Figures 1-8
Supplementary Tables 1-4

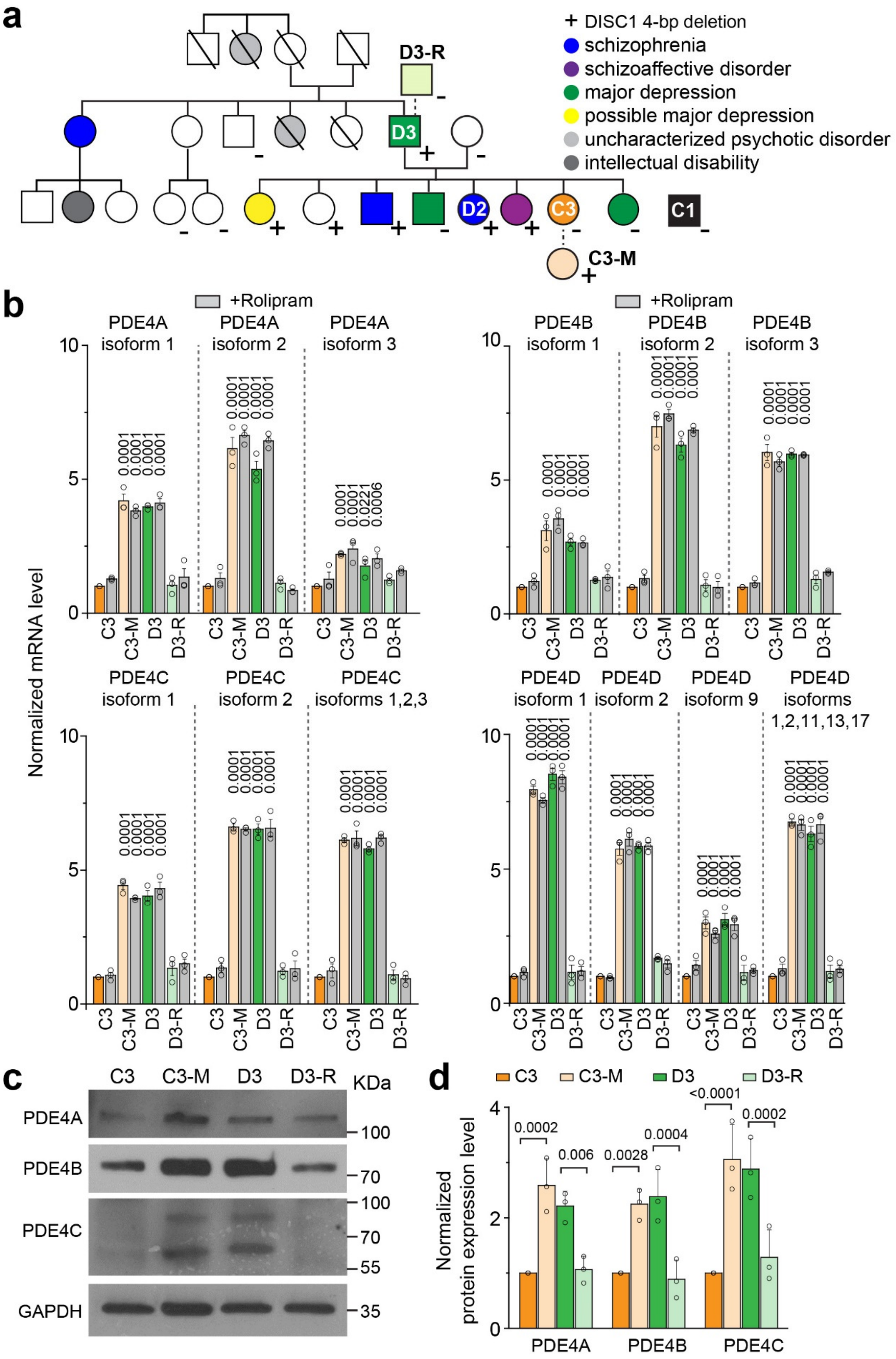

Supplementary Figure 1. Expression of PDE4 family members in forebrain cortical neurons derived from control and DISC1 mutant iPSC lines. a A schematic diagram of the Pedigree H

iPSC lines and isogenic mutant and rescue lines used in the current study. The pedigree has been updated and revised from the original study (Sachs et al. 2005). **b** RT-qPCR analysis for isoforms of PDE4A, PDE4B, PDE4C and PDE4D in 4-week human forebrain neurons upon vehicle or Rolipram (100 nM) treatment. Values represent mean ± s.e.m. (n = 3 independent cultures; each compared to its isogenic control using One-way ANOVA with p values indicated). **c-d** Protein expression level of PDE4s in neurons from different iPSC lines. Shown are sample western blot images (**c**) and quantification (**d**) of protein levels of PDE4A, PDE4B and PDE4C. Data were normalized to GAPDH for sample loading. Values represent mean ± s.d. (n = 3 independent cultures; One-way ANOVA with p values indicated). Source data are provided as a Source Data file.

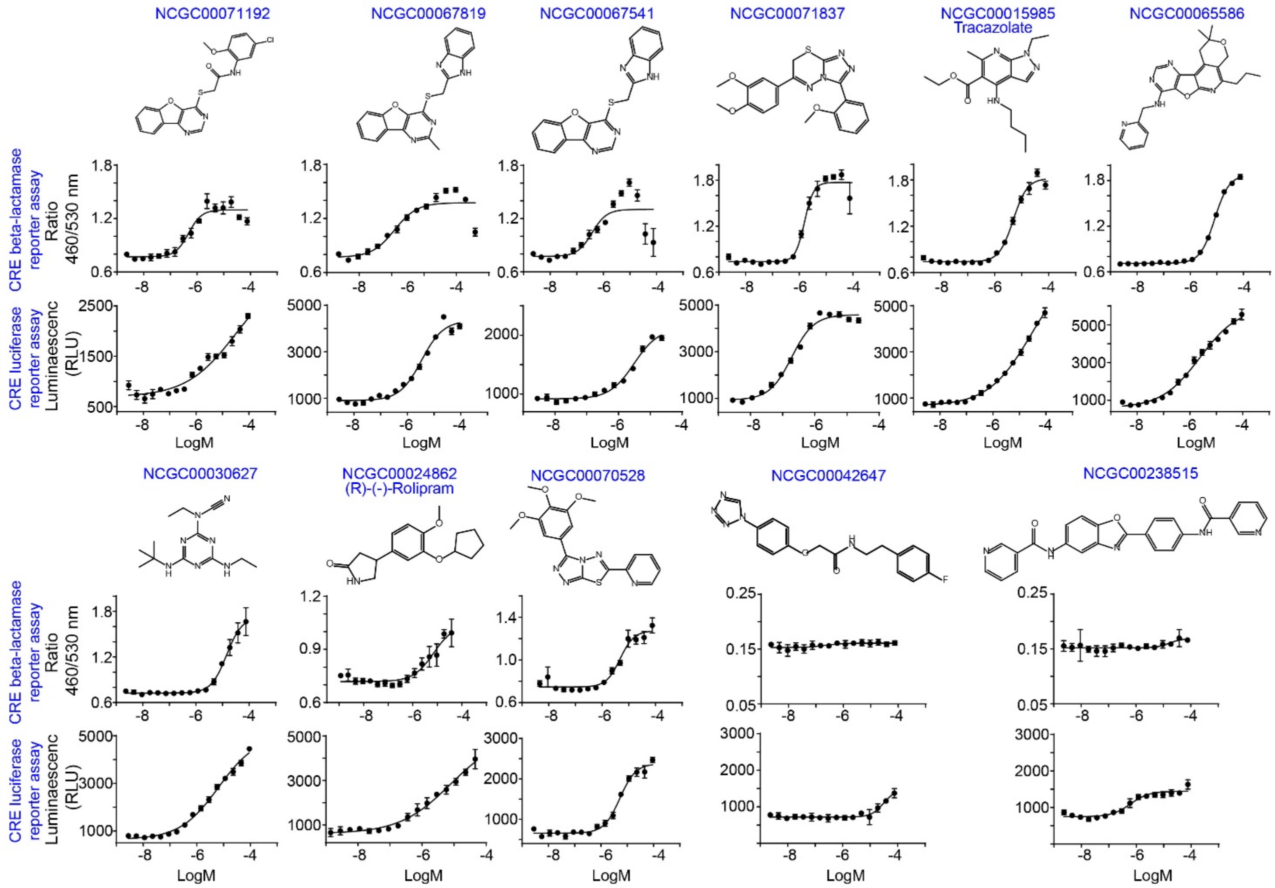

Supplementary Figure 2. Effect of a collection of 11 small molecules on cAMP-CREB activation. Shown are chemical structures (top), the CRE beta-lactamase reporter assay (middle) and the CRE luciferase reporter assay (bottom) of 11 small molecules for activating the CREB signaling pathway. Note minimal activity (< 12% efficacy) of NCGC00042674 and 00238515 at higher concentrations in the CRE luciferase reporter assay. Values represent mean $\pm$ s.d. (n = 4 independent wells). Source data are provided as a Source Data file.

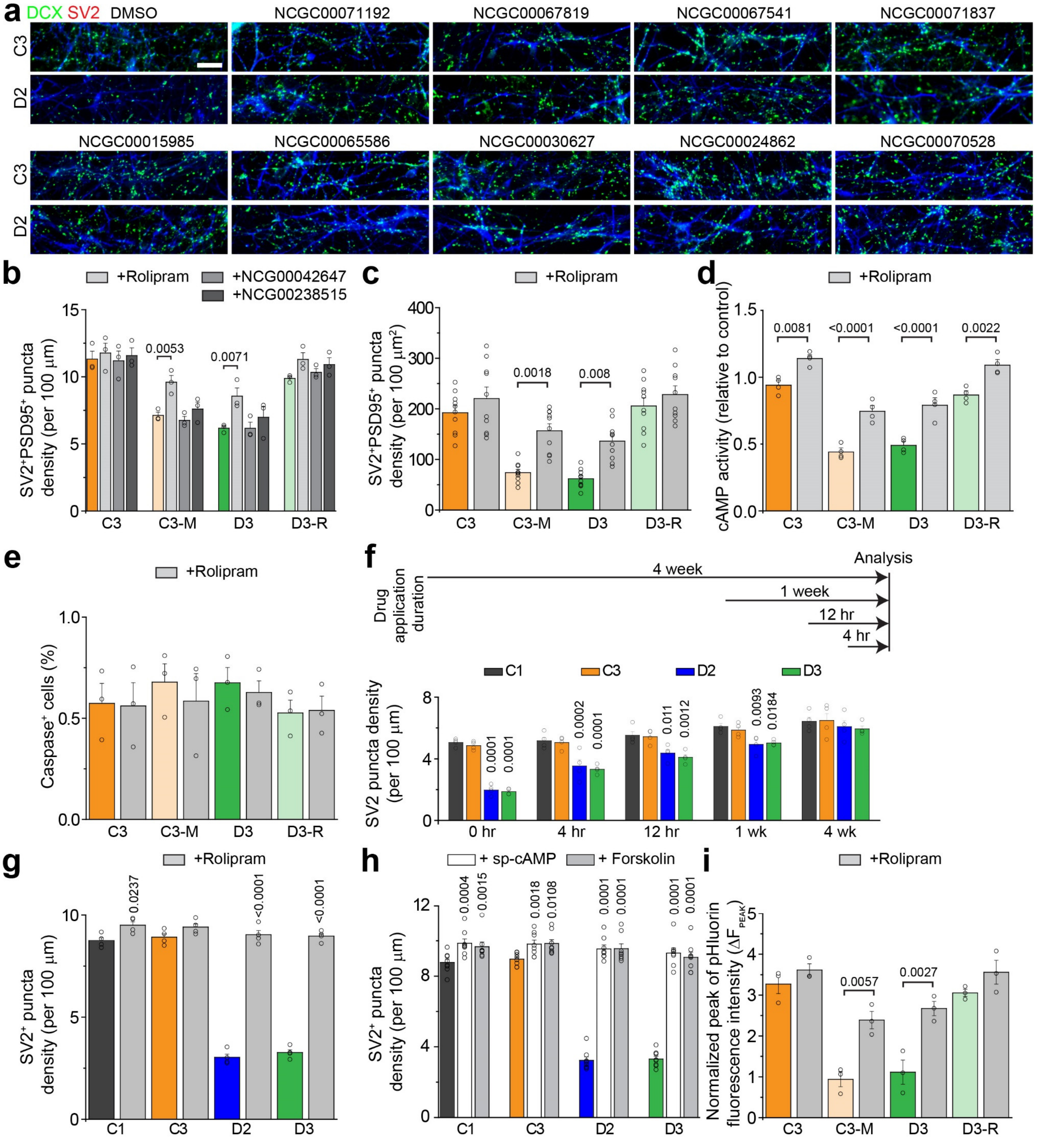

Supplementary Figure 3. Mechanism-guided pharmacological rescue of synaptic defects of human neurons carrying the DISC1 mutation. a Sample images of 6-week old neurons from C3 and D2 iPSC lines immunostained with SV2 and DCX under different treatment conditions. Scale bar, 20 µm. **b** Quantification of $SV2^+PSD95^+$ puncta density of neurons derived from different iPSC lines co-cultured with astrocytes at 4 weeks after neuronal differentiation upon vehicle, NCG00042647 (10 µM), NCG00238515 (10 µM) or Rolipram (100 nM) treatment. The values were normalized to the dendrite length (per 100 µm). Values represent mean ± s.e.m. (n = 3 independent

cultures; One-way ANOVA with p values indicated). **c** Quantification of $SV2^+PSD95^+$ puncta density of neurons derived from different iPSC lines co-cultured with astrocytes at 4 weeks after neuronal differentiation upon vehicle or Rolipram (100 nM) treatment. The values were normalized to the dendrite area (per 10^4 μm^2). The same cultures as in **Fig. 1f** were quantified. Values represent mean ± s.e.m. (n = 10 independent cultures; One-way ANOVA with p values indicated). **d** cAMP assay in human iPSC-derived cortical neurons upon vehicle or Rolipram (100 nM) treatment. Values represent mean ± s.e.m. (n = 4 independent cultures; One-way ANOVA with p values indicated). **e** Quantification of cleaved caspase3$^+$ cells in human iPSC-derived cortical neurons upon vehicle or Rolipram (100 nM) treatment. Values represent mean ± s.e.m. (n = 3 independent cultures). **f** An experimental scheme of drug treatment (upper) and quantification of the density of $SV2^+$ puncta of neurons derived from different iPSC lines at 4 weeks, with Rolipram (100 nM) treatment for different durations (lower). Values represent mean ± s.e.m. (n = 4 independent cultures; each compared to C1 using One-way ANOVA with p values indicated). **g** Summaries of quantification of $SV2^+$ puncta density of 6-week-old neurons upon vehicle or Rolipram (100 nM) treatment. Rolipram is present throughout the 6 week culture period. Values represent mean ± s.e.m. (n = 4 independent cultures; each compared to no drug treatment using One-way ANOVA with p values indicated). **h** Summaries of quantification of $SV2^+$ puncta density of 6-week-old neurons derived from different iPSC lines upon sp-cAMP (20 μM) or Forskolin (10 μM) treatment. Values represent mean ± s.e.m. (n = 8 independent cultures; each compared to no drug treatment using One-way ANOVA with p values indicated). **i** Summary of the normalized peak of Synapto-pHluorin fluorescence intensity (ΔF_{peak}) in response to the stimulation of 60 mM KCl in 4-week-old neurons upon vehicle or Rolipram (100 nM) treatment. Values represent mean ± s.e.m. (n = 3 independent differentiation cultures; One-way ANOVA with p values indicated). Source data are provided as a Source Data file.

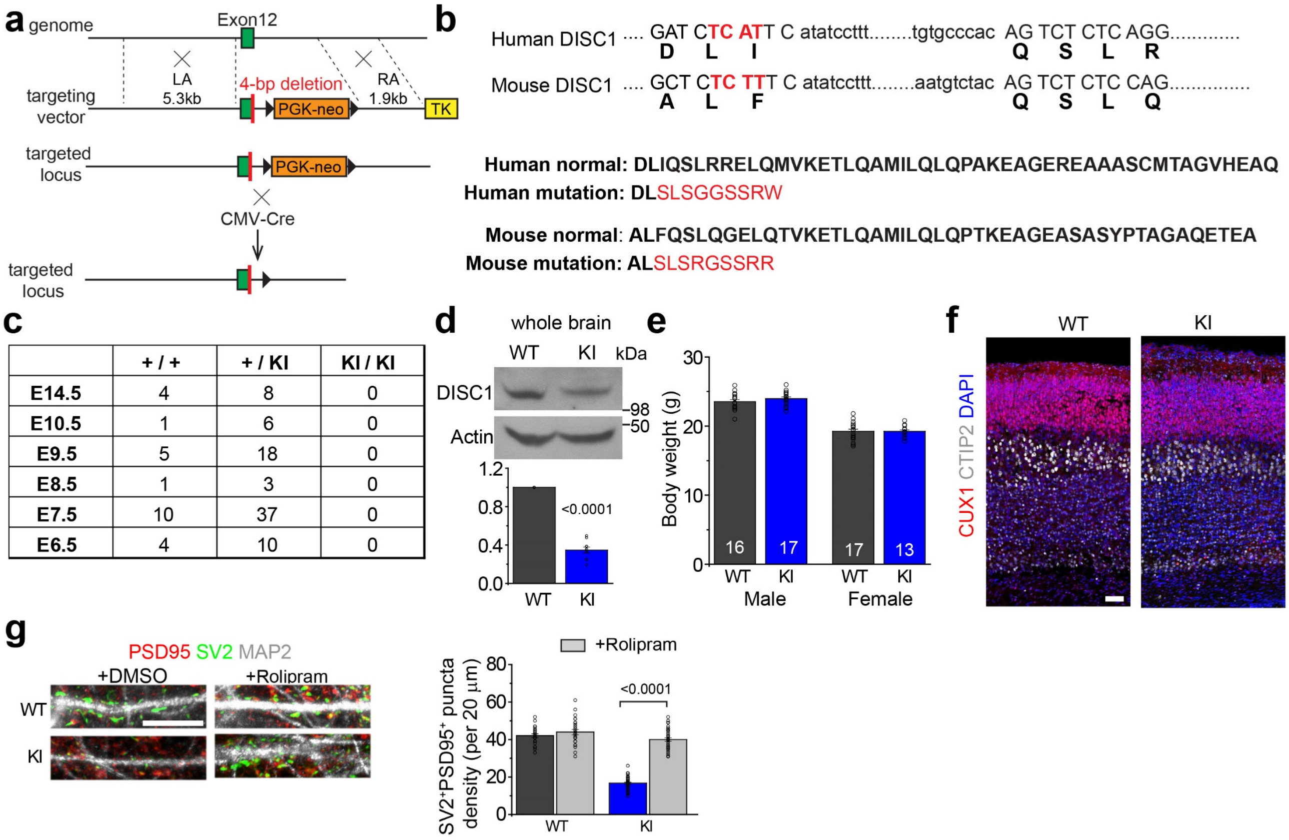

Supplementary Figure 4. Generation of Disc1 KI mouse carrying a human disease-relevant mutation in the Disc1 gene. a Targeting strategy for the generation of Disc1 KI mouse. **b** Predicted protein sequences at the C-terminus of mutant DISC1 in human and mouse. The deleted 4-bp are marked with red. **c** Homozygous Disc1 KI mice are early embryonic lethal. **d** Protein expression levels in whole brain lysates of 4-month-old WT and KI mice. Shown are sample western blot images of DISC1 protein levels (top) and quantification (bottom). Data were normalized to beta-actin for sample loading and then to WT in the same blot for comparison. Values represent mean $\pm$ s.d. (n = 10 independent experiments; One-way ANOVA with the p value indicated). **e** Normal body weight of 2-month-old male and female KI mice. Values represent mean $\pm$ s.e.m. (male: n = 16 for WT and n = 17 for KI; female: n = 17 for WT and n = 13 for KI). **f** Grossly normal cortical layers at P0 for KI mice as shown by layer-specific markers, including Ctip2 (a deep cortical layer marker) and Cux1 (a upper cortical layer marker), and DAPI. Scale bar, 50 µm. The representative images were taken from 3 independent experiments. **g** Sample images of primary cortical neurons from WT and KI mouse immunostained with SV2, PSD95 and MAP2 with or without Rolipram treatment (left). Scale bar, 10 µm. Summary of quantification of $SV2^+PSD95^+$ puncta density in the neurons (right). Values represent mean ± s.e.m. (n = 3 independent cultures; One-way ANOVA with the p values indicated). Source data are provided as a Source Data file.

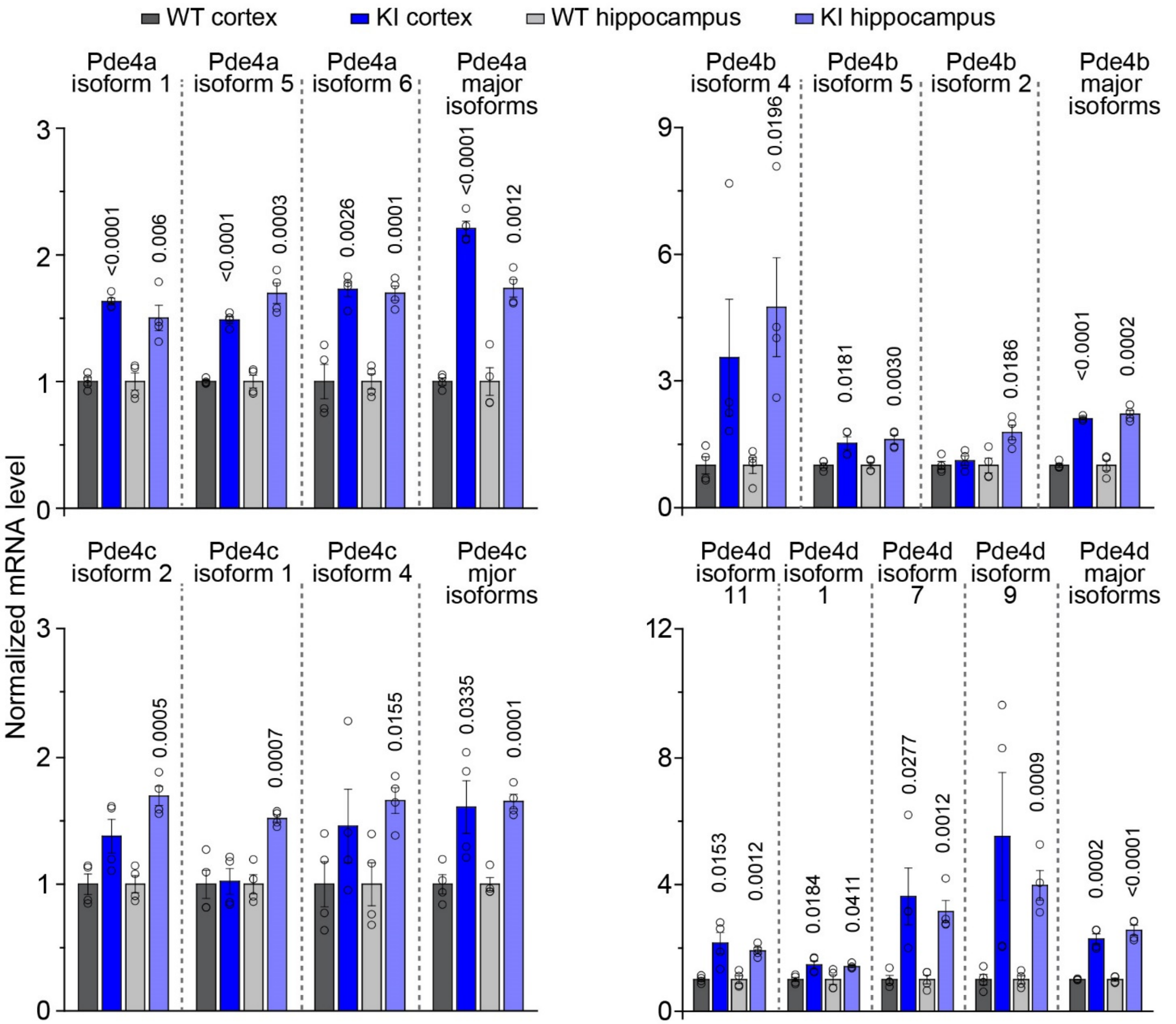

Supplementary Figure 5. Isoform expression of PDE4 family members in the WT and KI mouse cortex and hippocampus. RT-qPCR analysis for isoforms of Pde4a (top left), Pde4b (top right), Pde4c (bottom left), and Pde4d (bottom right) in cortical and hippocampal mRNA of WT and KI mice. Values represent mean ± s.e.m. (n = 4 biological replications from 2 independent animals; One-way ANOVA with p values indicated). Source data are provided as a Source Data file.

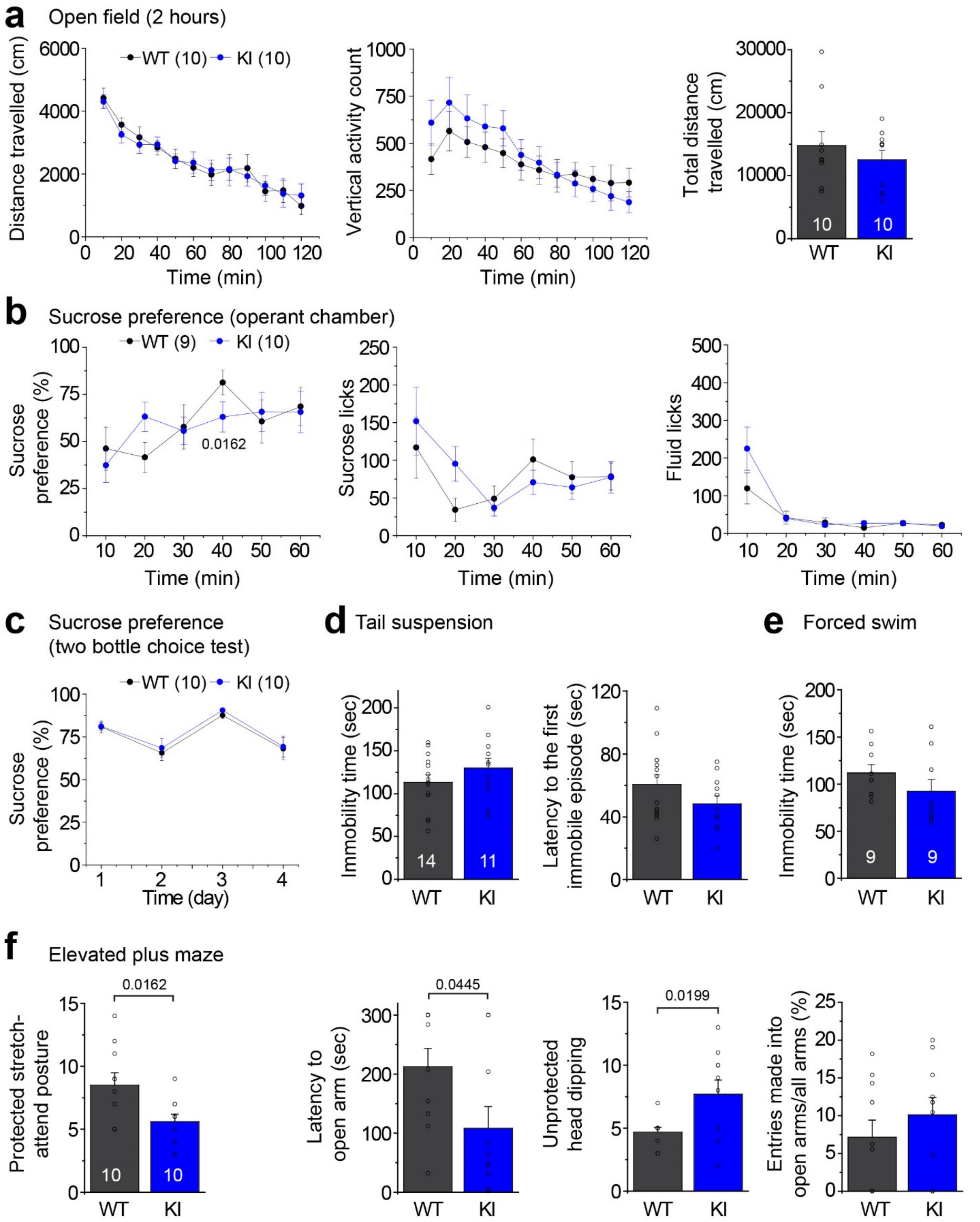

Supplementary Figure 6. Behavior analyses of Disc1 KI mice and WT littermates. a Basal locomotor activity was evaluated in the 2 hr open field test. Shown are total distance in 10-min bins (left), total vertical activity in 10-min bins (middle), and summary of total distance during 120 min

(right). Values represent mean ± s.e.m. (n = 10 for WT and KI, 10-week-old male littermates). **b** Sucrose preference test in operant conditioning chamber. Shown are percentage of total number of sucrose licks relative to the total number of fluid licks in a 10-min interval (left), the number of licks for sucrose for every 10 min (middle), and the number of fluid licks for every 10 min (right). Values represent mean ± s.e.m. (n = 9 for WT and n = 10 for KI, 12-week-old male littermates; One-way ANOVA with the p value indicated). **c** Two bottle choice sucrose preference test. Shown is the sucrose preference for each test day. Values represent mean ± s.e.m. (n = 10 for WT and n = 10 for KI, 12-week-old male littermates). **d** Tail suspension test. Mean duration of immobility of WT and KI mice (left). Mean latency time to the first immobile episode (right). Values represent mean ± s.e.m. (n = 14 for WT and n = 11 for KI, 15-week-old male littermates). **e** Forced swim test. Summary of duration of immobility of WT and KI mice in the water. Values represent mean ± s.e.m. (n = 9 for WT and KI, 20-22-week-old male littermates). **f** Elevated plus-maze test. The protected stretch-attend posture, latency to open arm entry, number of head dips, and the number of entries into open arms are calculated. Values represent mean ± s.e.m. (n = 10 for WT and KI, 10-week-old male littermates; One-way ANOVA with p values indicated). Source data are provided as a Source Data file.

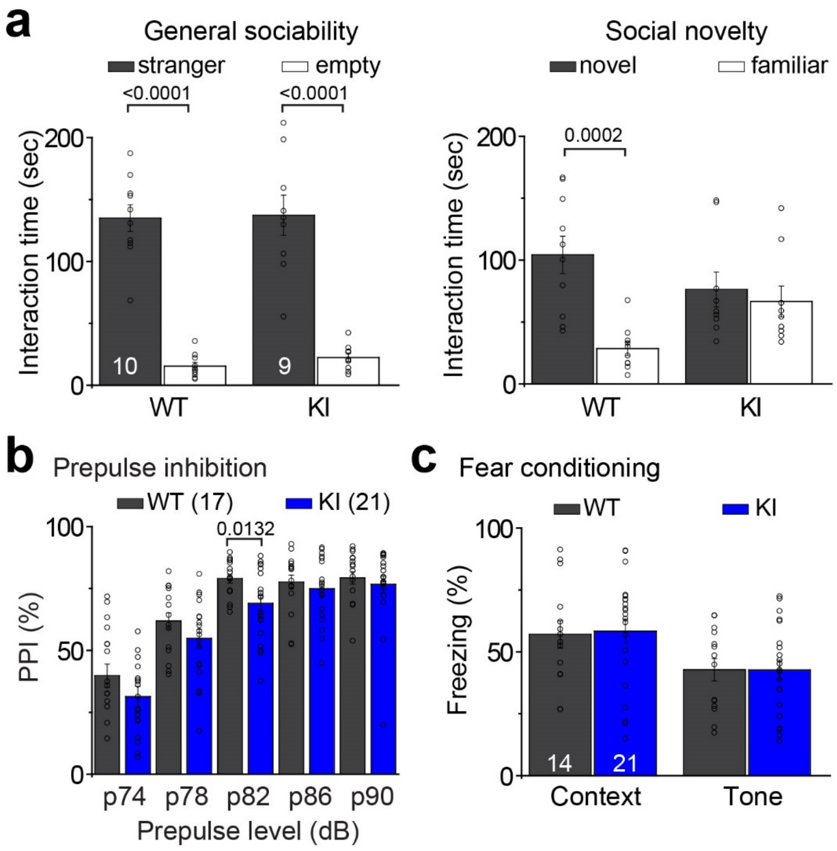

Supplementary Figure 7. Additional behavior analyses of Disc1 KI mice and WT littermates. **a** Three-chamber social preference test showing impaired social novelty recognition (novel versus familiar) of *Disc1* KI mice (right), but normal general sociability (left). Interaction time with familiar or novel mice spent in each chamber was calculated. Values represent mean ± s.e.m. (n = 10 for WT and n = 9 for KI, 16-week-old male littermates; One-way ANOVA with p values indicated). **b** Prepulse inhibition of acoustic startle response. PPI assay using a combination of startle (120 dB) and five prepulse levels (74 dB, 78 dB, 82 dB, 86 dB, and 90 dB) in WT and KI mice. Values represent mean ± s.e.m. (n = 17 for WT and n = 21 for KI, 22-24-week-old male littermates; One-way ANOVA with the p value indicated). **c** Context- and cue fear conditioning tests. Shown are percentages of freezing in each test. Values represent mean ± s.e.m. (n = 14 for WT and n = 21 for KI, 18-20-week-old male littermates). Source data are provided as a Source Data file.

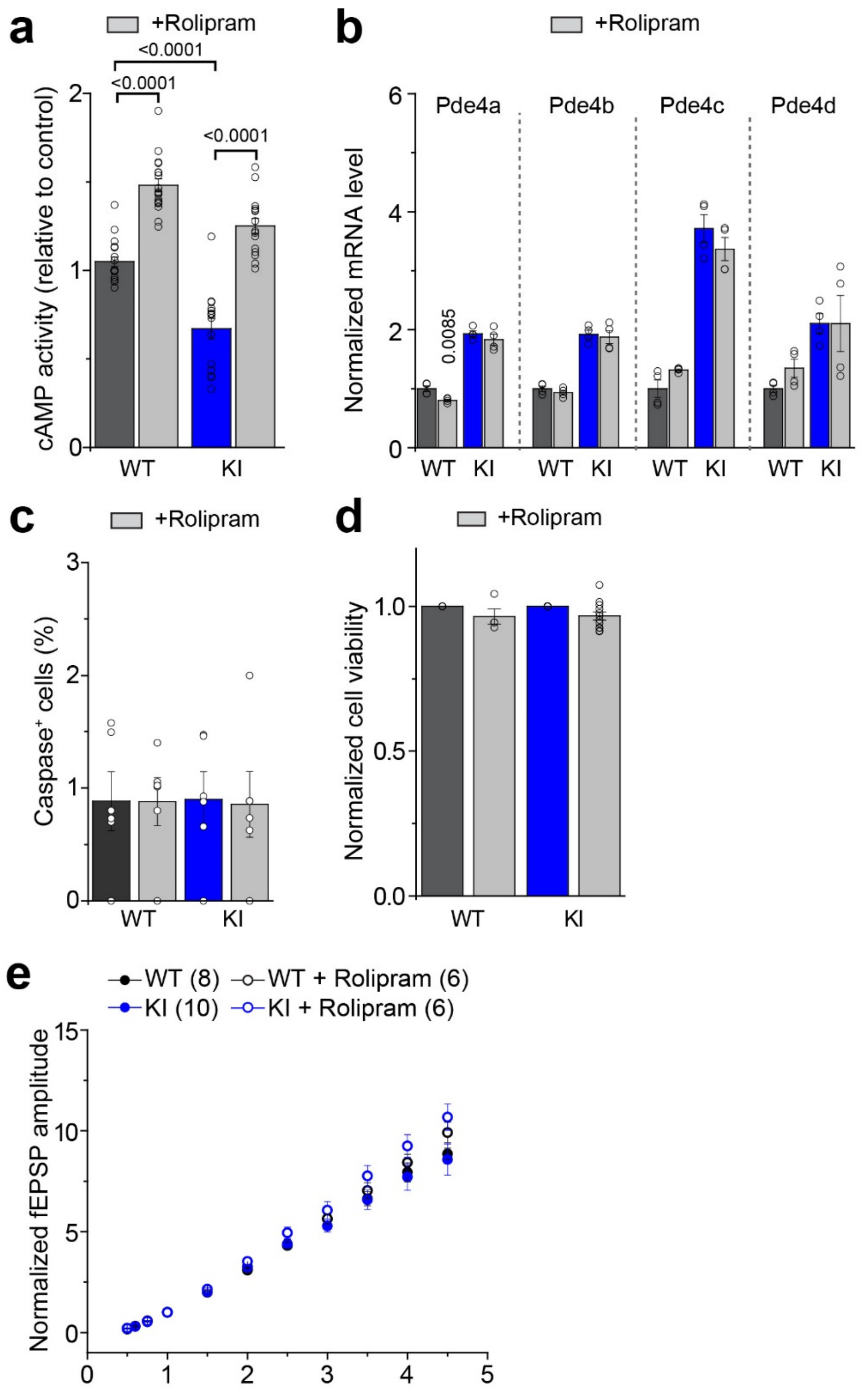

Supplementary Figure 8. Effect of Rolipram on Disc1 KI mice and WT littermates. a cAMP assay in primary cortical neurons of WT and KI mice with or without Rolipram (100 nM) treatment. Values represent mean ± s.e.m. (n = 18 cultures for WT and n = 15 cultures for KI; One-way ANOVA with the p value indicated). **b** RT-qPCR analysis of Pde4a, Pde4b, Pde4c and Pde4d in primary cortical neurons of WT and KI mice upon Rolipram (100 nM) treatment. Values represent mean ± s.e.m. (n = 4 independent cultures; $*p < 0.05$; One-way ANOVA). **c** Quantification of cleaved caspase^{+} cells in primary cortical neurons of WT and KI mice upon Rolipram (100 nM)

treatment. Values represent mean ± s.e.m. (n = 6 cultures). **d** Summary of cell viability assay in primary cortical neurons of WT and KI mice upon Rolipram (100 nM) treatment. Values represent mean ± s.e.m. (n = 4 for WT and n = 12 for KI cultures). **e** Electrophysiological analysis showed no changes in amplitude of fEPSPs of hippocampal slices upon Rolipram treatment. Rolipram (0.1mg/kg, i.p.) or vehicle was injected 30 min before sacrifice and was present in the bath solution throughout the recording. Values represent mean ± s.e.m. (n = numbers for each condition as indicated). Source data are provided as a Source Data file.

Supplementary Table 1. CRE reporter assay for the activity of each compound in activating the CREB signaling pathway

Compound Name	CRE beta-lactamase assay, EC_{50} (μM)	CRE-luciferase assay, EC_{50} (μM)	Concentrations used for human neurons based on EC_{50} values from two assays
NCGC00071192	0.5 ± 0.03	34.6 ± 4.31	2 μM
NCGC00067819	0.13 ± 0.01	3.78 ± 0.55	2 μM
NCGC00067541	0.41 ± 0.04	4.47 ± 1.93	2 μM
NCGC00071837	1.61 ± 0.06	0.2 ± 0.008	2 μM
NCGC00015985: Tracazolate	4.94 ± 0.10	22.2 ± 7.92	10 μM
NCGC00065586	8.97 ± 0.96	1.83 ± 0.76	10 μM
NCGC00030627	17.8 ± 4.95	6.93 ± 0.60	20 μM
NCGC00024862: (R)-(+)-Rolipram	7.32 ± 0.30	7.79 ± 0.04	20 μM
NCGC00070528-01	5.03 ± 0.37	5.08 ± 0.18	10 μM
NCGC00042647	Inactive	Minimal activity*	10 μM
NCGC00238515	Inactive	Minimal activity*	10 μM

*Note minimal activity (< 12% efficacy) of NCGC00042674 and 00238515 in the CRE-luciferase assay.

Supplementary Table 2. Summary information of antibodies used in the current study.

**Name**	**Host**	**Application**	**Dilution**	**Vendor**	**Catalog#**
Anti-ACTIN	Mouse	WB	1/10000	Sigma	A5316
Anti-CTIP2	Rat	ICH	1/500	abcam	ab18465
Anti-DCX	Goat	ICH	1/1000	Santa Cruz	sc-8066
Anti-DISC1	Rabbit	WB	1/1000	from Dr. Kaibuchi's lab	n/a
Anti-PDE4A	Rabbit	WB	1/1000	abcam	ab14607
Anti-PDE4B	Rabbit	WB	1/1000	abcam	ab14611
Anti-PDE4C	Rabbit	WB	1/1000	abcam	ab14608
Anti-SATB2	Mouse	ICH	1/500	abcam	ab92446
Anti-SV2	Mouse	WB	1/1000	DSHB	SV2
Anti-Cleaved caspase3	Rabbit	ICH	1/500	Cell Signaling	9661
Anti-MAP2	Chicken	ICH	1/500	Novus	nb300-213
Anti-PSD95	Rabbit	ICH	1/500	ThermoFisher Scientific	51-6900
Anti-GAPDH	Rabbit	WB	1/1000	Cell Signaling	5174
Horseradish peroxidase-conjugated anti-rabbit IgG	Goat	WB	1/7500	Santa Cruz	sc-2004
Horseradish peroxidase-conjugated anti-mouse IgG	Goat	WB	1/7500	Santa Cruz	sc-2005

Supplementary Table 3. Primer sequences used for each gene.

Gene Name	Gene ID (GI)	Application	Forward	Reverse
hPDE4A	162329606	qPCR	TTCTTCTGCGAGACCTGCTC	CGTTCCTTCATCGTGGGTGA
hPDE4A1	1519316441	qPCR	ATGCCATGGACACCAGCG	GATGGTGTCGGCCCATTCTC
hPDE4A2	162329609	qPCR	AACTTTCCGCAGACGCCTT	GATGGTGTCGGCCCATTCTC
hPDE4A3	162329611	qPCR	GATGAGGACACCCGTCGGC	TGTCATAGTCGCTGTCTGAGC
hPDE4B1	1519242941	qPCR	TCCGGGCAGACTTTCGTC	TCAATGCAGTTTGCTGACACTT
hPDE4B2	661903025	qPCR	TCCAGCCTAACTACATGCCT	GTGTGTCAGCTCCCGGTTC
hPDE4B3	661903026	qPCR	ATTTCTCCACGCAGTTCACCA	GATCCAGTGGACTCCGACCT
hPDE4C	341604763	qPCR	CAAGGCCATGTCTCGGAACT	TGGATGAAGGGTTTCCGACG
hPDE4C1	1054412301	qPCR	ATGGGACCCAGGCAGGAC	GTAGAAGGTGGAACTGGGGC
hPDE4C2	1054315330	qPCR	TACGGTGACCTTTGACCCAA	ACACGAGAGCCCATTTTCCA
hPDE4C1, 2, 3	1677500183	qPCR	CAAGGCCATGTCTCGGAACT	TGGATGAAGGGTTTCCGACG
hPDE4D	157277986	qPCR	GCAAGATCGAGCACCTAGCA	TCTAGCTGGTCCAGACACCA
hPDE4D1	1519312952	qPCR	CCTCCTCGTTCCAGGGACTCA	GAGGTCATAATCGCTGTCGGAT
hPDE4D2	157277986	qPCR	CCGGGCTAATTCTCCAAGCA	CAGACTGGCCAAGACCTGAG
hPDE4D9	1675071460	qPCR	GACCAATGTCTCAGATCAGTGG	GTCAAGGGCCGGTTACCAG
hPDE4D1,2, 11, 13, 17	1408287176	qPCR	GCAAGATCGAGCACCTAGCA	TCTAGCTGGTCCAGACACCA
hPDE4DIP	305410863	qPCR	CAGAAGGAGAGCATGGAACAG	ATGGTTCCTGGAAGGCAAG
hGAPDH	378404907	qPCR	TGGTCTCCTCTGACTTCAACAGCG	AGGGGTCTACATGGCAACTGTGAG
mpde4a	187952221	qPCR	TGCGACATCTTCCAGAACCT	ACCATGTTCCTGAGGACCTG
mpde4a_iso1	160333589	qPCR	TTCAAGTGCAGCCTCCAGTA	CCTGGGAATCAAACTGTGCT
mpde4a_iso5	160333586	qPCR	CCACTGAGCTCGGACAGG	CCGGGAAGAGAGAGAGACAG
mpde4a_iso6	894216310	qPCR	GTTTCACAGGGCTCTTCTGC	CCACACTTGTCGCGTCCTA
mpde4b	17225437	qPCR	CCTGCTGACGAACCTTCATG	CGGTAGGTCTGGATGGTCTC
mpde4b_iso4	295789132	qPCR	CCTGGATGACCTCCATTTGT	CAGACACCTGGTTCCCTGAT
mpde4b_iso5	295789136	qPCR	TGTTGCTGACGTTTCTCCAG	GGGTTGAGGGTGAGTCTTCA
mpde4b_iso2	295789128	qPCR	GTGACCCACCTGAAGCATTT	TCCCCCTCTTTGCTTTTCTT
mpde4c	21410862	qPCR	TGTGTTCAAAGTGGCTGAGC	TAGTGTCAGCAAGTACGCCA
mpde4c_iso2	118130951	qPCR	GCATCCAGCAGGGTGACCGG	CTTCCAAGGTGTCCAGAAGC
mpde4c_iso1	882939016	qPCR	CAAGCCGCTACCCCTCTAC	ATTTCTCCATCGAGGCTGTG
mpde4c_isoX4	1720426163	qPCR	CCCGGAGCATCTAAAAAGGT	AGCAGCTTCCTGAAGAGCAC
mpde4d	312922380	qPCR	GAGATTCTCGCGGCCATTTT	TGGTTCTCTAGGACCGAGGA
mpde4d_iso11	169742982	qPCR	TCTCGTACGGCGACTTTCTT	TCCACATCGAAACCACTTGA
mpde4d_iso1	110277929	qPCR	GACGTCAAGCTGGAGCATCT	ATGAGGGCTGCTCCTTCATA
mpde4d7	22901888	qPCR	CTCTTGCACCCCCATCAC	GCACGGCAGTACAGGTAGC
mpde4d9	37256027	qPCR	GACAACGGAGGCAGTTTGTT	CTTGGAGAATCAGCCCAGAC
mgapdh	148877869	qPCR	TCAACAGCAACTCCCACTCTTCCA	ACCCTGTTGCTGTAGCCGTATTCA
mDisc1	n/a	ESC screen	GGGAGGATTGGGAAGACAAT	CTGGACAGGGTCAAATGCTT
mDisc1	n/a	Genotyping in mouse	AGGCTTCACAGAGTCCTCCT	CAAACCTTCCCTCCCCTAAG

Supplementary Table 4. Summary of behavioral test results from various Disc1 mutant mouse models. N: normal; E: elevated; D: decreased; ND: not determined.

**Test**	**Parameter**	**Transgenic mouse models**			**Disc1 locus mutations in mouse**			**Knock-out mouse**	**Knock-in mouse**
		Hikida et al 2007 (Ref[1])	Li et al 2007 (Ref[2])	Pletnikov et al 2008 (Ref[3])	Koike et al 2006 (Ref[4]) Kvajo et al 2008 (Ref[5])	Clapcote et al 2007 (Ref[6])		Kuroda et al 2011 (ref[7])	Present study
						Q31L	L100P		
Open field	Total movement	E	N	E	N	N	E	N	N
Amphetamine-induced hyperactivity	Locomotor activity	ND	ND	E	ND	ND	E	ND	E
Elevated plus-maze	Time in open arm	N	ND	N	ND	N	N	E	E
Social interaction	Interaction time	N	D	D	ND	D	N	E	D
Y-maze	Time in new arm	ND	D	ND	D	ND	ND	N	D
T-maze	Alteration	N	ND	ND	ND	ND	ND	N	D
Novel object recognition	Exploration time	ND	ND	ND	ND	ND	ND	E	D
Forced swim/Tail suspension	Immobility time	E	E	E	ND	E	N	N	N
Sucrose	Sucrose preference	ND	ND	ND	ND	ND	ND	ND	N
Fear conditioning	Context-dependent freezing	ND	ND	ND	N	ND	ND	E	N
	Tone-dependent freezing	ND	ND	ND	N	ND	ND	N	N
PPI	Startle response	D	ND	N	N	D	D	D	N

Supplementary References:

1. Hikida, T., *et al.* Dominant-negative DISC1 transgenic mice display schizophrenia-associated phenotypes detected by measures translatable to humans. *Proc Natl Acad Sci U S A* **104**, 14501-14506 (2007).
2. Li, W., *et al.* Specific developmental disruption of disrupted-in-schizophrenia-1 function results in schizophrenia-related phenotypes in mice. *Proc Natl Acad Sci U S A* **104**, 18280-18285 (2007).
3. Pletnikov, M.V., *et al.* Inducible expression of mutant human DISC1 in mice is associated with brain and behavioral abnormalities reminiscent of schizophrenia. *Mol Psychiatry* **13**, 173-186, 115 (2008).
4. Koike, H., Arguello, P.A., Kvajo, M., Karayiorgou, M. & Gogos, J.A. Disc1 is mutated in the 129S6/SvEv strain and modulates working memory in mice. *Proc Natl Acad Sci U S A* **103**, 3693-3697 (2006).

5. Kvajo, M., *et al.* A mutation in mouse Disc1 that models a schizophrenia risk allele leads to specific alterations in neuronal architecture and cognition. *Proc Natl Acad Sci U S A* **105**, 7076-7081 (2008).
6. Clapcote, S.J., *et al.* Behavior phenotypes of Disc1 missense mutation in mice. *Neuron* **54**, 387-402 (2007).
7. Kuroda, K., *et al.* Behavioral alterations associated with targeted disruption of exons 2 and 3 of the Disc1 gene in the mouse. *Human molecular genetics* **20**, 4666-4683 (2011).
