# Supplementary material for: Pharmacological rescue in patient iPSC and mouse models with a rare DISC1 mutation
Source: Nat Commun. 2021 Mar 3;12:1398. doi: 10.1038/s41467-021-21713-3 (PMC7930023; doi:10.1038/s41467-021-21713-3)
Supplement: Supplementary file 2 — Reporting Summary [file 41467_2021_21713_MOESM2_ESM.pdf]

nature research

Corresponding author(s): Hongjun Song, Xin-yun Lu, and Guo-li Ming

Reporting Summary

Nature Research wishes to improve the reproducibility of the work that we publish. This form provides structure for consistency and transparency in reporting. For further information on Nature Research policies, see Authors & Referees and the Editorial Policy Checklist.

Statistical parameters

When statistical analyses are reported, confirm that the following items are present in the relevant location (e.g. figure legend, table legend, main text, or Methods section).

n/a	Confirmed	
☐	☒	The exact sample size (*n*) for each experimental group/condition, given as a discrete number and unit of measurement
☐	☒	An indication of whether measurements were taken from distinct samples or whether the same sample was measured repeatedly
☐	☒	The statistical test(s) used AND whether they are one- or two-sided<br>*Only common tests should be described solely by name; describe more complex techniques in the Methods section.*
☐	☒	A description of all covariates tested
☐	☒	A description of any assumptions or corrections, such as tests of normality and adjustment for multiple comparisons
☐	☒	A full description of the statistics including central tendency (e.g. means) or other basic estimates (e.g. regression coefficient) AND variation (e.g. standard deviation) or associated estimates of uncertainty (e.g. confidence intervals)
☐	☒	For null hypothesis testing, the test statistic (e.g. *F*, *t*, *r*) with confidence intervals, effect sizes, degrees of freedom and *P* value noted<br>*Give P values as exact values whenever suitable.*
☒	☐	For Bayesian analysis, information on the choice of priors and Markov chain Monte Carlo settings
☒	☐	For hierarchical and complex designs, identification of the appropriate level for tests and full reporting of outcomes
☒	☐	Estimates of effect sizes (e.g. Cohen's *d*, Pearson's *r*), indicating how they were calculated
☐	☒	Clearly defined error bars<br>*State explicitly what error bars represent (e.g. SD, SE, CI)*

Our web collection on statistics for biologists may be useful.

Software and code

Policy information about availability of computer code

Data collection	Image analysis - Image J with FIJI, version 1.49
Data analysis	locomotion activity - Fusion software (OmniTech Electronics, version 5.3)<br>Elevated plus maze, Y maze, Three-chamber social test - AnyMaze software (San Diego Instruments Inc., version 4.99)<br>Acoustic startle response and prepulse inhibition - SR-LAB software (San Diego Instruments Inc., version 6500-0091-L)<br>Electrophysiological analysis - pClamp 9 software (Molecular Devices, version 10.7), MiniAnalysis software (Synaptosoft, version 6.0)<br>Elevated plus maze, open arena test, Three-chamber social test, T-maze -EthoVision XT (Noldus Information Tech., version 7) & Nikon NIS-Elements Software (Nikon Instrument Inc., version 4.60.00)

For manuscripts utilizing custom algorithms or software that are central to the research but not yet described in published literature, software must be made available to editors/reviewers upon request. We strongly encourage code deposition in a community repository (e.g. GitHub). See the Nature Research guidelines for submitting code & software for further information.

Data

Policy information about availability of data

All manuscripts must include a data availability statement. This statement should provide the following information, where applicable:

- Accession codes, unique identifiers, or web links for publicly available datasets
- A list of figures that have associated raw data
- A description of any restrictions on data availability

The authors declare that all data supporting the findings of this study are available within the paper and its supplementary information files.

Field-specific reporting

Please select the best fit for your research. If you are not sure, read the appropriate sections before making your selection.

[x] Life sciences [ ] Behavioural & social sciences [ ] Ecological, evolutionary & environmental sciences

For a reference copy of the document with all sections, see nature.com/authors/policies/ReportingSummary-flat.pdf

Life sciences study design

All studies must disclose on these points even when the disclosure is negative.

Sample size	Sample size was determined based on our previous experience and the work of other groups using human iPSC-derived neurons and mouse as experimental model systems (Wen et al., Nature, 2014; Jang et al. Molecular Psychiatry, 2013). Sample sizes are reported in Figure legends or Figures.
Data exclusions	No data were excluded.
Replication	All the experimental data were replicated at least in three independent experiments. The experimental findings were reliably reproduced. Some behavioral experiments with mice were replicated with two different protocols and performed by different investigators in two institutes and yielding same conclusions.
Randomization	Samples were randomly allocated to control and experimental groups.
Blinding	Investigators involved in conducting experiments, collecting data, and performing analyses were blinded to the genotypes of iPSCs or mice.

Reporting for specific materials, systems and methods

Materials & experimental systems

n/a	Involved in the study	
[ ]	[x]	Unique biological materials
[ ]	[x]	Antibodies
[ ]	[x]	Eukaryotic cell lines
[x]	[ ]	Palaeontology
[ ]	[x]	Animals and other organisms
[x]	[ ]	Human research participants

Methods

n/a	Involved in the study	
[x]	[ ]	ChIP-seq
[x]	[ ]	Flow cytometry
[x]	[ ]	MRI-based neuroimaging

Unique biological materials

Policy information about availability of materials

Obtaining unique materials	The human iPSC lines we generated will be made available to researchers upon reasonable request. The mouse line (DISC1 KI) generated will be available from The JAX laboratory (Stock No. 036106).

Antibodies

Antibodies used	Antibody - Host species - Company - Catalog # - Western blot dilution - ICC/IHC dilution<br>Actin - Mouse - Sigma - A5316 - 1/10000 - n/a

CTIP2 - Rat - abcam - ab18465 - n/a - 1/500
DCX - Goat - Santa Cruz - sc-8066 -n/a - 1/1000
DISC1 - Rabbit - provided from Dr. Kaibuchi's lab - n/a - 1/2000 - n/a
GAPDH – Rabbit – Cell signaling – 5174 – 1:1000 – n/a
PDE4A - Rabbit - abcam - ab14607 - 1/1000 - n/a
PDE4B - Rabbit - abcam - ab14611 - 1/1000 - n/a
PDE4C - Rabbit - abcam - ab14608 - 1/1000- n/a
SATB2 - Mouse - abcam - ab92446 - n/a - 1/500
SV2 - Mouse - DSHB - sv2 - 1/1000 - 1/500
Cleaved Caspase3 - Rabbit - Cell Signaling - 9661 - n/a - 1/500
MAP2 - Chicken - Novus - nb300-213 - n/a - 1/500
PSD95 - Rabbit - ThermoFisher Scientific - 51-6900 - n/a - 1/500
Secondary antibody:
Horseradish peroxidase-conjugated anti-rabbit IgG - Goat - Santa Cruz Biotechnology - 31460 - 1:7500 - n/a
Horseradish peroxidase-conjugated anti-mouse IgG - Goat - Santa Cruz Biotechnology - 31430 - 1:7500 - n/a

Validation

All commercial antibodies were validated by manufacturers and by us as data provided in the manuscript.
The DISC1 antibody provided by Kaibuchi group has been validated in several published work and by us. Kuroda et al., Behavioral alterations associated with targeted disruption of exons 2 and 3 of the Disc1 gene in the mouse, Hum Mol Genet., 1;20 (23):4666-83, 2011

Eukaryotic cell lines

Policy information about cell lines

Cell line source(s)

Human induced pluripotent stem (iPS) cells from healthy donors and patients with mental disorders used for the current study were previously generated by our group and have been deposited at NINDS human cell and data repository (NHCDR) .
CHO CRE β-lactamase cell line -Invitrogen K1129 (now ThermoFisher K1535)
HEK293 CRE-luciferase cell line -Promega E8500

Authentication

The pluripotency of iPSC lines were confirmed by immunocytochemistry of pluripotency markers and in vivo teratoma assay into three germ layers. All of the tested iPSC lines have normal karyotypes.
STR profiling were done for HEK293 CRE-luciferase cells and confirmed to be 100% HEK cells.
CHO CRE β-lactamase cells were not authenticated.

Mycoplasma contamination

Mycoplasma contamination for iPSC cells were regularly tested, and all cell lines were negative for mycoplasma contamination.

Commonly misidentified lines
(See ICLAC register)

None of the cell lines used are listed in the ICLAC database.

Animals and other organisms

Policy information about studies involving animals; ARRIVE guidelines recommended for reporting animal research

Laboratory animals

All used mice were C57BL/6, male, adults (10-24 weeks old) or pregnant females . All mice were obtained from commercial vendors, except for the DISC1 mutant knockin mouse line we generated from mouse ESCs.
Mice were bred and maintained under specific pathogen-free conditions, and kept at an ambient temperature of 21°C and humidity of 40-60% under a 12-hour light/dark cycle with standard chow diet.

Wild animals

No wild animals were used in the study.

Field-collected samples

No field-collected samples were used in the study.
